# A review of current evidence about lncRNA MEG3: A tumor suppressor in multiple cancers

**DOI:** 10.3389/fcell.2022.997633

**Published:** 2022-12-05

**Authors:** Jie Xu, Xia Wang, Chunming Zhu, Kefeng Wang

**Affiliations:** ^1^ Department of Urology, Shengjing Hospital of China Medical University, Shenyang, China; ^2^ Department of Family Medicine, Shengjing Hospital of China Medical University, Shenyang, China

**Keywords:** long non-coding RNA, MEG3, tumor suppressor, cancer, diagnosis

## Abstract

Long non-coding RNA (lncRNA) maternally expressed gene 3 (MEG3) is a lncRNA located at the DLK1-MEG3 site of human chromosome 14q32.3. The expression of MEG3 in various tumors is substantially lower than that in normal adjacent tissues, and deletion of MEG3 expression is involved in the occurrence of many tumors. The high expression of MEG3 could inhibit the occurrence and development of tumors through several mechanisms, which has become a research hotspot in recent years. As a member of tumor suppressor lncRNAs, MEG3 is expected to be a new target for tumor diagnosis and treatment. This review discusses the molecular mechanisms of MEG3 in different tumors and future challenges for the diagnosis and treatment of cancers through MEG3.

## 1 Introduction

Cancer is a major global public health issue and the leading cause of death in the United States (Siegel et al., 2022). Since 1991, tumor mortality rates have continued to decline, resulting in estimated 3.2 million fewer deaths (Siegel et al., 2022). This has led to slow or stagnant research on cancers suitable for early screening, such as breast cancer (BC), prostate cancer (PCa), and colorectal cancer (CRC) (Siegel et al., 2022). Therefore, it is of great importance to strengthen research on antitumor mechanisms.

Long noncoding RNAs (LncRNAs) are a class of RNA molecules longer than 200 nucleotides that do not encode any proteins (Arun et al., 2016). Previously, lncRNAs were ignored as genomic transcription noises, but growing evidence shows that lncRNAs are a class of RNAs with special functions. LncRNAs are closely associated with the occurrence of human diseases (Bridges et al., 2021). Studies have found that many diseases, including cancers, are closely related to abnormal sequence and spatial structure, abnormal expression, and abnormal protein binding of lncRNAs (Liang et al., 2020). For example, lncRNA urothelial cancer associated 1 (UCA1) promotes PCa progression by sponging miR-143 (Yu et al., 2020a). In addition, the lncRNA zinc finger protein 24 transcription regulator (ZNFTR) plays an inhibitory role in pancreatic cancer (PC) by modulating the activating transcription factor (ATF3)/zinc finger protein 24 (ZNF24)/vascular endothelial growth factor A (VEGFA) pathway (Li et al., 2021). Although growing evidence shows the importance of lncRNAs in cancers, further studies are still needed.

**FIGURE 1:**
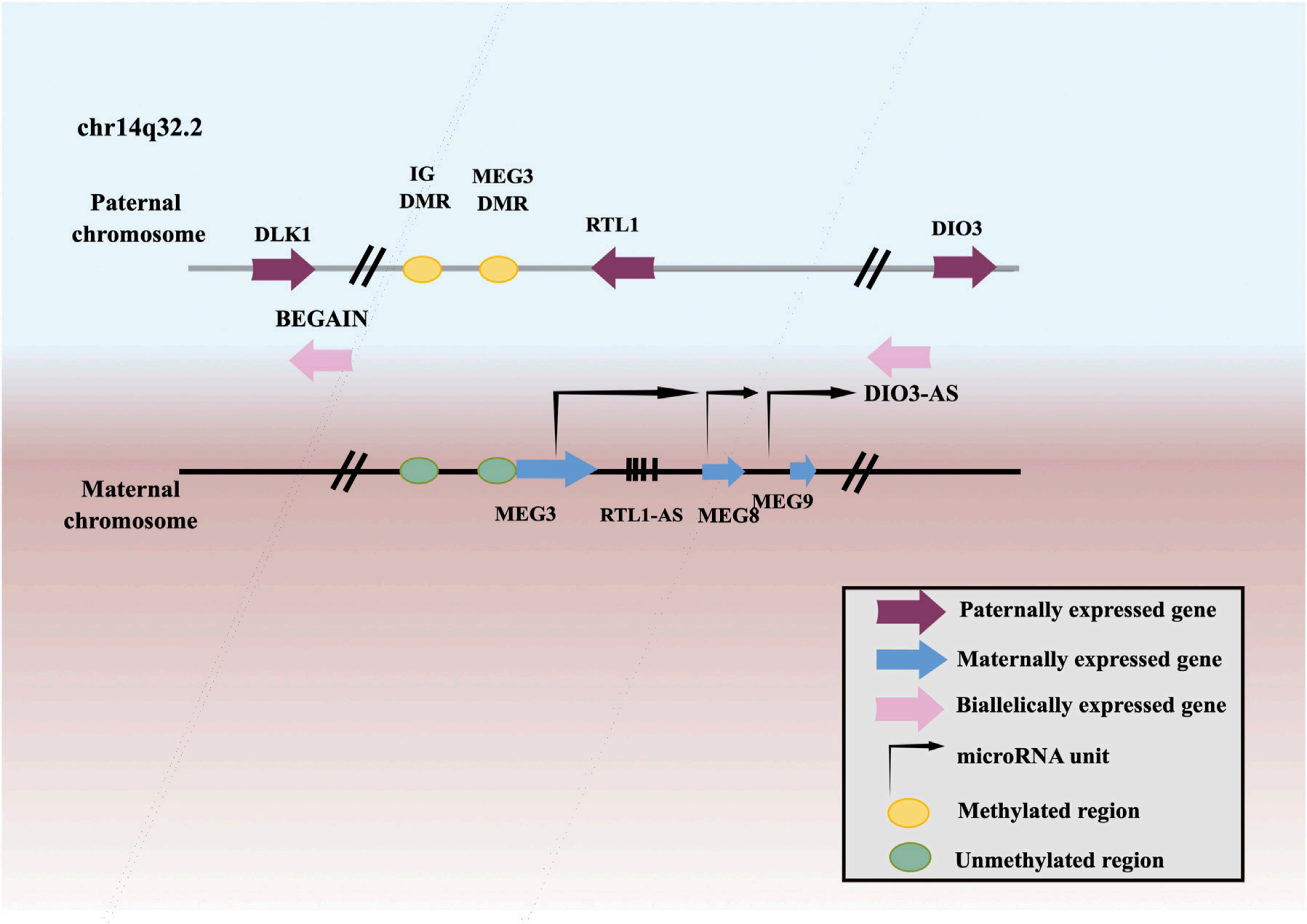
Schematic representation of the DLK1–MEG3 locus on human chromosome 14.

MEG3, a member of lncRNAs, is an imprinted gene that belongs to the DLK1-MEG3 imprinting region of human chromosome 14 and was first discovered as a homologous imprinted gene of mouse GLT2 (Figure 1). MEG3 has been found to be downregulated in many neoplasms, including squamous cell carcinoma of the head and neck (Ji et al., 2020), lung cancer (Zhao et al., 2019a), esophageal squamous cell carcinoma (ESCC) (Li et al., 2020a), gastric cancer (GC) (Yan et al., 2014), hepatocellular carcinoma (HCC) (He et al., 2017), gallbladder cancer (GBC) (Bao et al., 2020), cholangiocarcinoma (CCA) (Lu, 2019), CRC (Wang et al., 2021a), renal cell cancer (RCC) (He et al., 2018), Wilms’ tumor (WT) (Teng et al., 2020), bladder cancer (BCa) (Shan et al., 2020), PCa (Wu et al., 2019), testicular germ cell tumor (TGCT) (Yang et al., 2016), BC (Shaker et al., 2021), ovarian cancer (OC) (Tao et al., 2020), endometrial cancer (EC) (Xu et al., 2020), choriocarcinoma (Ji and Li, 2019), cervical cancer (CC) (Pan et al., 2021a), leukemia (Yu et al., 2020b), multiple myeloma (MM) (Shen et al., 2018), T-cell lymphoblastic lymphoma (T-LBL) (Fan et al., 2017), neuroblastoma (Ye et al., 2020), glioma (Qin et al., 2020), meningioma (Ding et al., 2020), retinoblastoma (RB) (Gao and Lu, 2016), thyroid cancer (TC) (Liu et al., 2018a), PC (Zhang and Feng, 2017), osteosarcoma (Shen et al., 2019), chordoma (Chen et al., 2017), melanoma (Wu et al., 2020). Low expression or deletion of MEG3 is associated with large tumor size, advanced FIGO stage, deep infiltration, early metastasis, and poor survival. Whereas, ectopic expression of MEG3 could inhibit the proliferation, migration, and invasion of tumor cells, and promote tumor cells apoptosis. Therefore, MEG3 is considered as a potential tumor suppressor.

## 2 MEG3 in various cancers

### 2.1 MEG3 in respiratory system neoplasms

#### 2.1.1 MEG3 in squamous cell carcinoma of the head and neck

Squamous cell carcinoma of the head and neck mainly includes squamous cell carcinoma of the tongue, nasopharyngeal carcinoma (NPC), laryngeal carcinoma, and oral carcinoma. Squamous cell carcinoma of the head and neck is a leading cause of cancer and death. More than 600,000 cases are diagnosed worldwide annually. It is highly aggressive, has a short survival period, and is prone to chemotherapy resistance (Ferris et al., 2016). Therefore, it is crucial to identify new treatment strategies.

Jia et al. (2014) reported that the upregulated expression level of MEG3 suppressed the proliferation and cell cycle progression as well as promoted cell apoptosis in tongue squamous cell carcinoma cells. However, the limitation of this study is that the experimental data failed to prove the expression level of miR-26a alone as a prognostic marker of tongue squamous cell carcinoma. A team from the Chinese University of Hong Kong confirmed that MEG3 acts as a tumor suppressor in NPC (Chak et al., 2017). Therefore, it is necessary to conduct in-depth studies to promote the development of lncRNA-oriented diagnosis and treatment strategies for NPC. Further, Zhao et al. (2019b) found that high expression of MEG3 inhibited the proliferation, migration, and invasion of laryngeal cancer cells, as well as the epithelial-to-mesenchymal transition (EMT) process. A recent study revealed that the expression of MEG3 was downregulated in oral squamous cell carcinoma and the overexpression of MEG3 negatively regulated the Wnt/β-catenin signaling pathway to inhibit the proliferation and metastasis of tumor cells (Liu et al., 2017). One year later, another group from Chongqing Medical University suggested that MEG3 may regulate the proliferation and metastasis of oral squamous cell carcinoma cells by targeting miR-21 (Zhang et al., 2018). Subsequently, another study pointed out that rs11160608 of the MEG3 gene is related to the occurrence of oral squamous cell carcinoma in the Chinese population (Hou et al., 2019). However, this paper lacks large sample size and experimental research.

Many studies have also elucidated lncRNA-miRNA-mRNA networks in squamous cell carcinoma of the head and neck. Emerging evidence elucidated that MEG3 inhibited the EMT process by targeting E-cadherin and reducing the expression of miR-421 (Ji et al., 2020). A study by Lin et al. (2021) showed that MEG3 can promote autophagy and apoptosis in NPC cells by increasing phosphatase and tensin homolog (PTEN) expression through interaction with miR-21. Further, another study pointed out that MEG3 regulated the expression of apoptotic peptidase activating factor 1 (APAF-1) by competitively binding to miR-23a in laryngeal cancer (Zhang et al., 2019a). In addition, Tan et al. (2019) found that high expression of MEG3 promotes the expression of suppressor of cytokine signaling (SOCS) 5 and SOCS6 by inhibiting the expression of miR-548d-3p, thus regulating the JAK-STAT signaling pathway in oral cancer. Based on this, further studies on the characterization and downstream signaling pathways of MEG3 will help to develop novel therapeutic strategies. An in-depth study confirmed that MEG3 inhibits the stemness and invasion of oral tumor stem cells by sponging miR-421 (Chen et al., 2021).

In summary, MEG3 plays a tumor suppressor role in various head and neck malignancies through lncRNAs-miRNAs-mRNAs regulatory pathway and some other mechanisms. These studies provide new ideas for the clinical treatment of squamous cell carcinoma of the head and neck. However, the sample size of some of these experimental studies is not large enough and the exploration of the exact molecular mechanism is lacking, which needs to be improved in future studies.

#### 2.1.2 MEG3 in lung cancer

Lung neoplasms are one of the four major tumors (Siegel et al., 2022). In line with pathological features, lung neoplasms consist of non-small cell lung cancer (NSCLC) and small cell lung cancer. Among them, NSCLC occupies approximately 85% of lung tumor cases (Yang et al., 2011). However, because symptoms are not obvious in the early stage, most patients with NSCLC are not diagnosed until the late stage, resulting in a poor prognosis (Lai et al., 2012). Therefore, early diagnosis and effective treatment of lung neoplasms remains a crucial area that needs to be studied.

Chemotherapy resistance is a main factor that affects the prognosis of patients with lung neoplasms. MEG3 up-regulates the sensitivity of lung tumor cells to chemotherapy drugs. Recently, it was reported that MEG3 enhances the anti-tumor activity of curcumin in gemcitabine-resistant NSCLC cells through the PTEN pathway (Gao et al., 2021). In addition, Xia et al. (2015) and Wang et al. (2017a) demonstrated that downregulation of the expression level of MEG3 enhances cisplatin resistance of lung tumor cells by regulating the WNT/β-catenin signaling pathway and the miR-21-5p/SRY-box transcription factor 7 (SOX7) axis, respectively. Moreover, a group from Jilin University clarified that overexpression of MEG3 inhibited autophagy of tumor cells, thus improving the sensitivity of vincristine (Xia et al., 2018). Another study showed that MEG3 regulated mitochondrial apoptosis pathway induced by p53 and Bcl-xl to improve the cisplatin sensitivity of lung cancer to chemotherapy (Liu et al., 2015).

MEG3 has also been shown to act as a miRNA sponge in lung cancer. Su et al. (2016) has shown that MEG3 and miR-3163 might synergistically inhibit the translation of s-phase kinase-associated protein 2 (Skp2) mRNA in NSCLC, thus inhibiting the growth of NSCLC cells. Although this paper identified miR-3163 as a mediator of MEG3 regulation of Skp2, the possibility that Skp2 levels are altered by other mechanisms in NSCLC cells cannot be ruled out, and this possibility should be analyzed in future studies. One year later, another research group demonstrated that MEG3 regulates the miR-21-5p/SOX7 axis in cisplatin-resistant NSCLC cells (Wang et al., 2017a). In addition, Wu et al. (2018) proposed that MEG3 could reduce the degradation of BRCA1 by miR-7-5p and promote the apoptosis of NSCLC cells. Similar results elucidated that the MEG3/miR-650/solute carrier family 34-member 2 (SLC34A2) axis may be associated with stem cell morphology, cell migration and invasion (Zhao et al., 2019a). Furthermore, Lv et al. (2021) indicated that MEG3 inhibited NSCLC cell migration and invasion by sponging miR-21-5p, thereby upregulating PTEN expression. Another research group showed that the expression of MEG3 was decreased in NSCLC and was effective in the treatment of NSCLC by regulating miR-543/IDO signaling pathway to affect the immunity and autophagy of lung neoplasm cells (Wang et al., 2021b).

MEG3 also acts as a tumor suppressor through several other mechanisms or pathways. A recent study concluded that the MEG3 rs4081134 polymorphism is associated with a reduced risk of lung tumors in northeast China (Yang et al., 2018). Another significant research reported that the expression level of MEG3 in normal lung tissues was higher than that in NSCLC tissues, and the downregulation of MEG3 might be an adverse prognostic factor in patients with NSCLC (Zhang et al., 2017a). However, this study also has many shortcomings, such as this is a retrospective study, there are selection bias and non-randomization problems. Kruer et al. (2016) indicated that MEG3 assists in controlling the occurrence of lung cancer cells *via* the Rb pathway. Intriguingly, the innovation of this study is that it suggests that MEG3 is a downstream effector molecule of Rb pathway in inhibiting the progression of NSCLC. Subsequent studies have shown that MEG3 inhibits telomere activity and function, cell proliferation, migration, and invasion by moderating dyskerin pseudouridine synthase 1 protein expression, thus inhibiting the progression of NSCLC (Yang et al., 2020a). Similar results showed that upregulation of MEG3 activates p53, inhibits the proliferation of NSCLC cells, and partially promotes cell apoptosis (Xu et al., 2018a). And the next step is to study the specific regulatory mechanism of MEG3 by PTX in nude mice.

These experimental data suggest that MEG3 can be used as a diagnostic and therapeutic target for lung cancer by enhancing sensitivity to chemotherapy, acting as a molecular sponge, and other mechanisms. Besides, more large sample sizes and further studies are needed in the future to verify the existing conclusion.

It was demonstrated that MEG3 can inhibit the progress of respiratory system neoplasms through increasing the expression of E-cadherin, PTEN, APAF-1, JAK-STAT, Skp2, SOX7, BRCA1, SLC34A2, and IDO by sponging miR-421, miR-21-5p, miR-23a, miR-548d-3p, miR-3163, miR-7-5p, miR-650, and miR-543, respectively. The specific mechanisms and functional characteristics of MEG3 in respiratory system neoplasms are listed in Tables 1, 2.

**TABLE 1:** Characterization of MEG3 function in respiratory and digestive system neoplasms.

Tumor types	Expression	Role	Function role	miRNAs	Related gene	Biomarker type	References
Head and neck squamous cell carcinoma	Downregulation	Tumor suppressor	Proliferation, migration, invasion, and EMT	miR-421	E-cadherin	Diagnostic	Ji et al. (2020)
Nasopharyngeal carcinoma	Downregulation	Tumor suppressor	Autophagy	miR-21	PTEN	Diagnostic/Prognostic	Lin et al. (2021)
Laryngeal cancer	Downregulation	Tumor suppressor	Proliferation and apoptosis	miR-23a	APAF-1	Diagnostic/Prognostic	Zhang et al. (2019a)
Oral squamous cell	Downregulation	Tumor suppressor	Proliferation and migration	miR-21	—	Diagnostic/Prognostic	Zhang et al. (2018)
Oral squamous cell	Downregulation	Tumor suppressor	Migration and apoptosis	miR-548d-3p	JAK-STAT	Prognostic/Therapeutic	Tan et al. (2019)
Oral squamous cell	Downregulation	Tumor suppressor	Invasion	miR-421	—	Diagnostic	Chen et al. (2021)
Lung cancer	Downregulation	Tumor suppressor	Proliferation, apoptosis, and chemosensitivity	miR-21-5p	SOX7	Diagnostic	Wang et al. (2017a)
Lung cancer	Downregulation	Tumor suppressor	Proliferation	miR-3163	Skp2	Therapeutic	Su et al. (2016)
Lung cancer	Downregulation	Tumor suppressor	Apoptosis	miR-7-5p	BRCA1	Diagnostic	Wu et al. (2018)
Lung cancer	Downregulation	Tumor suppressor	Migration and invasion	miR-650	SLC34A2	Therapeutic	Zhao et al. (2019a)
Lung cancer	Downregulation	Tumor suppressor	Migration and invasion	miR-21-5p	PTEN	Therapeutic	Lv et al. (2021)
Lung cancer	Downregulation	Tumor suppressor	Immunity and autophagy	miR-543	IDO	Therapeutic	Wang et al. (2021b)
Gastric cancer	Downregulation	Tumor suppressor	Proliferation	miR-148a	DNMT-1	Therapeutic	Yan et al. (2014)
Gastric cancer	Downregulation	Tumor suppressor	Proliferation, invasion, and migration	miR-181a	Bcl-2	Diagnostic/Therapeutic	Peng et al. (2015)
Gastric cancer	Downregulation	Tumor suppressor	Proliferation and apoptosis	miR-141	E2F3	Therapeutic	Zhou et al. (2015)
Gastric cancer	Downregulation	Tumor suppressor	Proliferation and invasion	miR-208a	SFRP1	Therapeutic	Cui et al. (2018a)
Gastric cancer	Downregulation	Tumor suppressor	Proliferation, migration, invasion, and apoptosis	miR-181a-5p	ATP4B	Diagnostic/Therapeutic	Ding et al. (2019)
Gastric cancer	Downregulation	Tumor suppressor	Proliferation and metastasis	miR-21	—	Therapeutic	Dan et al. (2018)
Gastric cancer	Downregulation	Tumor suppressor	Proliferation and invasion	miR-770	—	Prognostic	Guo et al. (2017)
Gastric cancer	Downregulation	Tumor suppressor	Proliferation, metastasis, and EMT	miR-21	—	Therapeutic	Xu et al. (2018b)
Hepatocellular carcinoma	Downregulation	Tumor suppressor	Proliferation and apoptosis	miR-29	—	Therapeutic	Braconi et al. (2011)
Hepatocellular carcinoma	Downregulation	Tumor suppressor	Proliferation, invasion, and migration	miR-26a	DNMT3B	Therapeutic	Li et al. (2017)
Hepatocellular carcinoma	Downregulation	Tumor suppressor	Proliferation and colony formation	miR-122	PKM2, PTEN	Prognostic/Therapeutic	Zheng et al. (2018)
Hepatocellular carcinoma	Downregulation	Tumor suppressor	Proliferation and colony formation	miR-664	ADH4	Diagnostic	He et al. (2017)
Hepatocellular carcinoma	Downregulation	Tumor suppressor	Proliferation and migration	miR-483-3p	ERp29	Diagnostic/Prognostic	Li et al. (2019a)
Hepatocellular carcinoma	Downregulation	Tumor suppressor	Proliferation and apoptosis	miR-9-5p	SOX11	Diagnostic/Therapeutic	Liu et al. (2019)
Hepatocellular carcinoma	Downregulation	Tumor suppressor	Proliferation, migration, and invasion	miR-10a-5p	PTEN/AKT/MMP-2/MMP-9	Therapeutic	Zhang et al. (2019b)
Hepatocellular carcinoma	Downregulation	Tumor suppressor	Proliferation, migration, and invasion	miR-544b	BTG2	Therapeutic	Wu et al. (2021)
Cholangiocarcinoma	Downregulation	Tumor suppressor	Cell viability and apoptosis	miR-361-5p	TRAF3	Therapeutic	Lu, (2019)
Colorectal cancer	Downregulation	Tumor suppressor	Chemosensitivity	miR-141	PDCD4	Therapeutic	Wang et al. (2018a)
Colorectal cancer	Downregulation	Tumor suppressor	Proliferation, invasion, and ER stress	miR-103a-3p	PDHB	Diagnostic/Prognostic	Wang et al. (2021a)

**TABLE 2:** Features of the studies included in this review of respiratory and digestive system neoplasms.

Study	Tumor types	Sample size (Normal: Tumor)	Detection method	*p* Value	TNM (*p* value)	LNM (*p* value)	DM (*p* value)	OS (*p* value)	References
Ji	Head and neck squamous cell carcinoma	(51:51)	qRT-PCR	*p* < 0.05	—	—	—	—	Ji et al. (2020)
Jia	Tongue squamous cell carcinoma	(76:76)	qRT-PCR	*p* < 0.05	—	—	—	—	Jia et al. (2014)
Lin	Nasopharyngeal carcinoma	(80:80)	qRT-PCR	*p* < 0.05	—	—	—	—	Lin et al. (2021)
Zhang	Laryngeal cancer	(50:50)	qRT-PCR	*p* < 0.05	—	—	—	—	Zhang et al. (2019a)
Zhao	Laryngeal cancer	(35:35)	qRT-PCR	*p* < 0.05	*p* = 0.015	*p* = 0.036	—	—	Zhao et al. (2019b)
Liu	Oral squamous cell	(83:83)	qRT-PCR	*p* < 0.05	—	—	—	—	Liu et al. (2017)
Zhang	Oral squamous cell	(45:45)	qRT-PCR	*p* < 0.05	—	—	—	—	Zhang et al. (2018)
Hou	Oral squamous cell	(444:984)	qRT-PCR	*p* = 0.017	—	—	—	—	Hou et al. (2019)
Wang	Lung cancer	(46:46)	qRT-PCR	*p* < 0.05	—	—	—	—	Wang et al. (2017a)
Liu	Lung cancer	(41:41)	qRT-PCR	*p* < 0.05	—	—	—	—	Liu et al. (2015)
Su	Lung cancer	(20:20)	qRT-PCR	*p* < 0.05	—	—	—	—	Su et al. (2016)
Wu	Lung cancer	(72:72)	qRT-PCR	*p* < 0.05	—	—	—	—	Wu et al. (2018)
Wang	Lung cancer	(78:69)	qRT-PCR	*p* < 0.05	—	—	—	—	Wang et al. (2021b)
Yang	Lung cancer	(526:526)	qRT-PCR	*p* < 0.05	—	—	—	—	Yang et al. (2018)
Zhang	Lung cancer	(1,144:1,144)	qRT-PCR	*p* = 0.007	—	—	—	*p* = 0.025	Zhang et al. (2017a)
Cui	Gastric cancer	(62:62)	qRT-PCR	*p* < 0.05	*p* = 0.018	*p* = 0.014	—	*p* < 0.05	Cui et al. (2018a)
Ding	Gastric cancer	(30:30)	qRT-PCR	*p* < 0.01	—	—	—	—	Ding et al. (2019)
Guo	Gastric cancer	(134:134)	qRT-PCR	*p* < 0.01	*p* = 0.001	*p* = 0.013	*p* = 0.01	*p* < 0.05	Guo et al. (2017)
Sun	Gastric cancer	(72:72)	qRT-PCR	*p* < 0.001	*p* = 0.022	*p* = 0.071	*p* = 0.775	—	Sun et al. (2014)
Wei	Gastric cancer	(31:31)	qRT-PCR	*p* < 0.05	—	—	—	—	Wei and Wang, (2017)
Kong	Gastric cancer	(474:543)	qRT-PCR	*p* = 0.002	—	—	—	—	Kong et al. (2020)
Zhu	Hepatocellular carcinoma	(23:23)	qRT-PCR	*p* < 0.05	—	—	—	—	Zhu et al. (2015)
Zhuo	Hepatocellular carcinoma	(72:72)	qRT-PCR	*p* < 0.05	*p* < 0.05	—	—	—	Zhuo et al. (2016)
Dong	Hepatocellular carcinoma	(54:54)	qRT-PCR	*p* < 0.01	—	—	—	*p* < 0.05	Dong et al. (2019)
Sun	Hepatocellular carcinoma	(72:72)	qRT-PCR	*p* < 0.01	—	—	—	—	Sun et al. (2019)
Li	Hepatocellular carcinoma	(46:46)	qRT-PCR	*p* < 0.01	*p* < 0.05	*p* < 0.05	*p* < 0.05	*p* < 0.05	Li et al. (2017)
Li	Hepatocellular carcinoma	(7:14)	qRT-PCR	*p* < 0.05	*p* = 0.104	—	*p* = 0.001	—	Li et al. (2019a)
Liu	Hepatocellular carcinoma	(30:30)	qRT-PCR	*p* < 0.01	*p* = 0.028	—	*p* < 0.001	—	Liu et al. (2019)
Liu	Gallbladder cancer	(84:84)	qRT-PCR	*p* < 0.05	—	—	—	—	Liu et al. (2016b)
Jin	Gallbladder cancer	(50:50)	qRT-PCR	*p* < 0.05	*p* = 0.047	*p* = 0.018	—	—	Jin et al. (2018)
Li	Cholangiocarcinoma	(60:60)	qRT-PCR	*p* < 0.01	*p* = 0.004	*p* = 0.032	—	—	Li et al. (2019b)
Lu	Cholangiocarcinoma	(20:20)	qRT-PCR	*p* < 0.05	—	—	—	—	Lu, (2019)
Yin	Colorectal cancer	(62:62)	qRT-PCR	*p* < 0.001	*p* = 0.037	*p* = 0.020	*p* = 0.004	—	Yin et al. (2015)
Cao	Colorectal cancer	(518:527)	qRT-PCR	*p* < 0.001	—	—	—	—	Cao et al. (2016)
Dong	Colorectal cancer	(84:84)	qRT-PCR	*p* < 0.05	—	—	—	—	Dong et al. (2018)
Wang	Colorectal cancer	(48:48)	qRT-PCR	*p* < 0.05	*p* = 0.042	*p* = 0.020	*p* = 0.637	*p* = 0.0213	Wang et al. (2018a)
Wang	Colorectal cancer	(42:42)	qRT-PCR	*p* < 0.01	*p* = 0.001	*p* = 0.236	—	*p* < 0.001	Wang et al. (2019a)
Zuo	Colorectal cancer	(80:80)	qRT-PCR	*p* < 0.05	—	—	—	—	Zuo et al. (2020)
Wang	Colorectal cancer	(33:33)	qRT-PCR	*p* < 0.05	—	—	—	—	Wang et al. (2021a)

### 2.2 MEG3 in digestive system neoplasms

#### 2.2.1 MEG3 in esophageal squamous cell carcinoma

ESCC is a familial aggregation of malignant cancers related to people’s eating habits. It is prevalent in some regions, particularly in counties bordering Hebei, Henan and Shanxi provinces in northern China. The molecular mechanism underlying ESCC remains unclear and requires further study.

The study by Dong et al. (2017) revealed that MEG3 might upregulate the expression levels of E-cadherin and forkhead box O1 (FOXO1) through sponging miR-9. Not long after, a study reported that the expression of MEG3 was downregulated in ESCC tissues, and that MEG3 might inhibit the growth of ESCC cells as well as induce apoptosis by activating endoplasmic reticulum (ER) stress (Huang et al., 2017). Another study generated similar results MEG3 was associated with tumor progression and poor prognosis (Ma et al., 2019). Additionally, Li et al. (2020a) reported that MEG3 inhibited EMT in ESCC by inhibiting the phosphoserine aminotransferase 1 (PSAT-1)-dependent glycogen synthase kinase 3 beta (GSK-3β)/Snail signaling pathway. A team from Nanchang University conducted an in-depth study on the mechanism of MEG3/MDM2/p53/miR-149-3p/forkhead box P3 (FOXP3) axis in ESCC occurrence and development (Xu et al., 2021a). Together, these data indicate that MEG3 serves as a tumor suppressor in ESCC.

#### 2.2.2 MEG3 in gastric cancer

The incidence of GC is the result of genetic and environmental factors and is associated with high mortality and metastasis rates. Among the top five most common cancers worldwide, GC ranks fourth and is the second leading cause of cancer-related deaths (Fock, 2014). Typically, GC is detected at an advanced stage of infection, proliferation, and lymph node metastasis. Gastrectomy remains the main treatment for GC, but patients with advanced GC cannot receive effective treatment (Catalano et al., 2009). Hence, it is imperative to understand GC’s underlying molecular mechanisms and to develop new treatments.

Recent studies have also showed that MEG3 plays a role as a tumor suppressor through the miRNA sponge mechanism. An in-depth study revealed that MEG3 partially inhibited the proliferation of GC cells by increasing DNA methyltransferase 1 (DNMT-1) by antagonizing miR-148a (Yan et al., 2014). One year later, another group confirmed that MEG3 upregulated Bcl-2 by competitively binding to miR-181a, thus suppressing the proliferation, migration, and invasion of GC cells (Peng et al., 2015). Similar results indicated that MEG3 might interact with miR-141 and target transcription factor 3 (E2F3) (Zhou et al., 2015). In addition, Cui et al. (2018a) found that MEG3 suppresses the progression of GC by targeting secreted frizzled-related protein 1 (SFRP1) and negatively regulating miR-208a. Besides, Ding et al. (2019) proved that MEG3 might appear to be a tumor suppressor by modulating the miR-181a-5p/ATP4B axis in GC.

Emerging evidence revealed that overexpression of MEG3 noticeably inhibited GC growth and metastasis by targeting miR-21 (Dan et al., 2018). Another research group found that miR-770 and its host gene MEG3 might play a tumor-suppressive role in GC (Guo et al., 2017). In addition, Sun et al. (2014) demonstrated that decreased expression of MEG3 in GC cells might be related to MEG3 hypermethylation. Upon restoring MEG3 expression or inhibiting methylation, tumor development can be inhibited both *in vivo* and *in vitro*. Subsequently, another research group revealed that the upregulated expression of MEG3 might function as a tumor suppressor gene by activating p53 (Wei and Wang, 2017). Further, Jiao and Zhang (2019) and Xu et al. (2018b) explained that MEG3 suppresses the growth and metastasis of GC cells by inhibiting EMT. A team from Nanjing Medical University identified that the MEG3 RS7158663 might be associated with the risk of gastric neoplasm (Kong et al., 2020).

Accordingly, these findings suggest that MEG3 acts as a tumor suppressor in GC through the miRNA sponge mechanism and other pathways. At present, the role of MEG3 in GC through enhancing the sensitivity of chemoradiotherapy is still blank, and researchers can strengthen the research in this aspect. Based on the existing mechanism, other molecular mechanisms of MEG3 in GC can be explored in the future.

#### 2.2.3 MEG3 in hepatocellular carcinoma

Undoubtedly, liver neoplasms pose a considerable threat to human health, and their incidence is increasing worldwide. HCC is the most common type of liver neoplasm, comprising approximately 90% of the cases (Llovet et al., 2021); its incidence is expected to increase due to the rise in hepatitis C cirrhosis and non-alcoholic fatty liver disease. Therefore, there is an urgent need to develop new strategies for treating liver cancer.

A research group highlighted that the tissue-specific regulation of miR-29a in MEG3 methylation in HCC cells might contribute to the growth of HCC (Braconi et al., 2011). A study by Anwar et al. (2012) confirmed that DLK1-MEG3 is absent in human HCC, thus, MEG3 is used as a biomarker to predict the outcome of epigenetic therapy. Three years later, another group confirmed that methylation of the MEG3 promoter region resulted in the downregulation of MEG3 in HCC cells. Further, dendritic curcumin can induce the re-expression of MEG3, offering a promising treatment for HCC (Zamani et al., 2015). In addition, a study by Zhu et al. (2015) found that MEG3 interacts with the p53 protein and activates its target gene, thus acting as a tumor suppressor in HCC cells. Notably, the innovation of this study is to reveal the interaction between MEG3 and p53 for the first time. Other researchers discovered that MEG3 can inhibit cell proliferation, invasion, colony formation, block the cell cycle, and promote apoptosis of HCC cells (Chang et al., 2016). Chen et al. (2016) confirmed that the tumor suppressive function of MEG3 was partly accomplished through the activation of ER stress and the p53 pathway. In addition, emerging evidence demonstrated that the ubiquitin-like with PHD and ring finger domains 1 (uHRF1)/DNMT1/MEG3/p53 axis might serve as a potential prognostic marker and therapeutic target for HCC (Zhuo et al., 2016). Concurrently, Liu et al. (2016a) reported that hypermethylation in the promoter region explained the low expression of MEG3 in HCC as MEG3 induces apoptosis by affecting cell cycle progression. In another study, Fan et al. (2019) demonstrated the molecular mechanism by which the arsenic trioxide PKM2 pathway regulating MEG3 in HCC by affecting EMT. Recently, it was reported that the downregulated expression of MEG3 might boost the proliferation, migration, and invasion of HCC by signaling the expression of TGF-β1 (Dong et al., 2019). Moreover, an in-depth study validated that downregulation of MEG3 promotes the proliferation and invasion ability of HCC cells, and the mechanism was related to the activation of the PI3K/AKT pathway by consistent adaptor-related protein complex 1 subunit gramma 1 (AP1G1) expression (Sun et al., 2019). In addition, Pu et al. (2019) innovatively proposed that MEG3 inhibited autophagy by downregulating interleukin enhancer binding factor 3 (ILF3) and regulating the PI3K-Akt-mTOR and Beclin-1 signaling pathways. Notably, this study suggested that low concentration of adenosine combined with ectopic expression of MEG3 may be a good therapeutic strategy for HCC. Recently, it was reported that MEG3 suppressed the development of HCC stem cells both *in vivo* and *in vitro* (Jiang et al., 2020).

Recently, many studies have elucidated lncRNA-miRNA-mRNA networks in HCC. Li et al. (2017) found that the molecular regulatory axis MEG3/miR-26a/DNA methyltransferase 3 beta (DNMT3B) is a promising new target for HCC treatment. Another study showed that MEG3 exerts anticancer effects in HCC by negatively regulating PKM2 and PTEN activities through miR-122 (Zheng et al., 2018). Similar results indicated that the overexpression of MEG3 in HCC partially released the inhibition of miR-664 on alcohol dehydrogenase 4 (ADH4) transcription and translation by sponging miR-664 (He et al., 2017). A team from Tianjin Medical University found that high glucose levels might improve the poor outcome of patients with HCC and diabetes by targeting the MEG3/miR-483-3p/ER protein 29 (ERp29) regulatory network (Li et al., 2019a). In the same year, another group proved that MEG3 was decreased in HCC tissues and cells, and its high expression regulates the miR-9-5p/SRY-box transcription factor 11 (SOX11) axis (Liu et al., 2019). Recently, it was reported that MEG3 regulates the PTEN/AKT/matrix metallopeptidase 2 (MMP-2)/matrix metallopeptidase 9 (MMP-9) signaling axis and participates in HCC development by targeting miR-10a-5p (Zhang et al., 2019b). In addition, Wu et al. (2021) showed that the MEG3/miR-544b/BTG2 axis may play a crucial role in the occurrence and progression of HCC.

MEG3 has been studied more in HCC than in other cancers. These studies fully demonstrate the role of MEG3 as a tumor suppressor in HCC. However, in these studies, some of the research teams still have some problems, such as insufficient sample size, lack of *in vivo* experimental verification, lack of further exploration of the downstream molecular mechanism of MEG3 and so on.

#### 2.2.4 MEG3 in gallbladder cancer

GBC is a highly fatal neoplasm and the most common cancer of the biliary system (Zhu et al., 2022). Therefore, it is important to elucidate the molecular mechanism of GBC.

Liu et al. (2016b) demonstrated that MEG3 was downregulated in GBC tissues, and MEG3 inhibited GBC cell proliferation and induce apoptosis. Two years later, another group reported that MEG3 inhibits the expression of the downstream gene, large tumor suppressor kinase 2 (LATS2), by regulating the stability of EZH2 (Jin et al., 2018). A study by Bao et al. (2020) showed that MEG3 inhibited cell proliferation and induced apoptosis in human GBC cells *in vitro*. Therefore, targeting MEG3 could be a new approach to GBC treatment.

#### 2.2.5 MEG3 in cholangiocarcinoma

CCA is a highly invasive and heterogeneous primary malignant tumor originating from the intrahepatic and extrahepatic bile duct epithelial cells. Most CCA are detected at a late stage when treatment is no longer effective (Liu et al., 2022). Therefore, an understanding of CCA’s molecular mechanisms and a search for new biomarkers could have a tremendous impact on CCA treatment.

A recent study demonstrated that CCA progression was largely suppressed by MEG3 as elevated expression of MEG3 inhibits cell viability, metastasis, and EMT (Li et al., 2019b). In addition, Lu (2019) found that MEG3 suppressed the occurrence and development of CCA by regulating miR-361-5p/TNF receptor-associated factor 3 (TRAF3)/NF-κB pathway.

#### 2.2.6 MEG3 in colorectal cancer

Despite great achievements in the prevention and treatment of CRC, the disease remains an important cause of cancer death, thus a global public health concern (Siegel et al., 2022). In addition, CRC’s pathogenesis is unknown, and the treatment course is long. Therefore, revealing its mechanism of onset and development may provide new therapeutic methods for improving patient prognosis.

Yin et al. (2015) reported that there was a significant positive correlation between low MEG3 expression and low histological grade, deep invasion, and advanced TNM stage in CRC tissues. A subsequent study showed that the rs7158663 single nucleotide polymorphism of MEG3 played a role in the CRC development (Cao et al., 2016). Moreover, an in-depth research demonstrated that decreased MEG3 expression may enhance CRC cell proliferation and inhibit apoptosis through upregulation of TGF-β1 and its downstream sphingosine kinase 1 (SPHK1) (Dong et al., 2018). It is important to note that this study used three different ethnic colorectal cancer cells, excluding racial differences in the pathogenesis of colorectal cancer, so the conclusion are relatively reliable. Subsequent studies showed that through the regulation of miR-141/programmed cell death 4 (PDCD4), MEG3 induces oxaliplatin sensitivity in CRC cells (Wang et al., 2018a). A study by Zhu et al. (2018) confirmed that MEG3 might inhibit the proliferation and metastasis of CRC cells by downregulating the expression level of Clusterin and its direct binding with the Clu protein. A team from China Medical University showed that MEG3 is downregulated in CRC and that it regulates cell function by targeting ADAR1 (Wang et al., 2019a). An in-depth study firstly document the correlation between MEG3 and serum vitamin D. and reported that MEG3 is a possible prognostic factor and therapeutic target for CRC (Zuo et al., 2020). Another study pointed out that the MEG3/miR-103a-3p/pyruvate dehydrogenase E1 subunit beta (PDHB) pathway regulates cell proliferation, invasion, and ER stress, thus confirming the antitumor effects of abnormal expression of MEG3 and miR-103a-3p gene knockout in CRC progression (Wang et al., 2021a).

It was demonstrated that MEG3 can inhibit the progress of digestive system neoplasms through increasing the expression of DNMT-1, Bcl-2, E2F3, SFRP1, ATP4B, DNMT3B, PKM2, PTEN, ADH4, ERp29, SOX11, AKT/MMP-2/MMP-9, BTG2, TRAF3, PDCD4, and PDHB by sponging miR-148a, miR-181a, miR-141, miR-208a, miR-26a, miR-122, miR-664, miR-483-3p, miR-9-5p, miR-10a-5p, miR-544b, miR-361-5p, and miR-103a-3p, respectively. The specific mechanisms and functional characteristics of MEG3 in digestive system neoplasms are listed in Tables 1, 2.

### 2.3 MEG3 in genitourinary system neoplasms

#### 2.3.1 MEG3 in renal cell cancer

Renal cancer is an extremely common form of malignant tumor of the urinary system and is mostly seen in men aged 60–70 years. With the expansion of conventional imaging examinations, the misdiagnosis rate has decreased (Capitanio and Montorsi, 2016) and the incidence of RCC is increasing. Therefore, RCC’s pathogenesis should be further explored to identify new biomarkers and therapeutic targets.

A recent study revealed that MEG3 activates the mitochondrial pathway by inhibiting the expression of Bcl-2, thus inducing apoptosis in RCC cells (Wang et al., 2015a). Subsequent studies showed that MEG3 upregulates the expression of RAS, such as family 11 member B (RASL11B), by downregulating the expression of miR-7 (He et al., 2018). In addition, Gong et al. (2020) showed that MEG3 suppressed tumor growth, and the MEG3/ST3Gal1/EGFR axis provides a new idea for the diagnosis and therapy of RCC. In summary, these data suggest that MEG3 is involved in RCC tumor suppression.

#### 2.3.2 MEG3 in Wilms’ tumor

Nephroblastoma, also known as WT, is the most prevalent primary kidney neoplasm in children, comprising 5% of overall in childhood cancers (Balis et al., 2021). Thus, understanding the pathogenesis of WT is important to improve patient prognosis.

The unprecedented results of Teng et al. (2020) proved that there is a significant reduction in MEG3 expression in WT tissues and cell lines. The overexpression of MEG3 dramatically decreased the growth, migration, and invasion of WT cells by modulating the Wnt/β-catenin pathway. In summary, this study demonstrated that MEG3 has an antitumor effect in WT.

#### 2.3.3 MEG3 in bladder cancer

As the most prevalent malignancy of the genitourinary system, BCa occurs at an alarming rate. In addition, the pathophysiological mechanisms underlying BCa remain unclear. Therefore, novel treatments are required.

Emerging evidence confirmed that the expression level of MEG3 in BCa tissues was dramatically reduced, whereas overexpression of MEG3 led to a reduction in autophagy ability in BCa (Ying et al., 2013). Similarly, Greife et al. (2014) reported that the expression of DLK1 and MEG3 generally decreased in BCa tissues and cell lines. A study by Jilin University showed that high expression of MEG3 inhibits the migration and invasion of BCa cells and enhance chemotherapy sensitivity to cisplatin (Feng et al., 2018). Another study pointed out that MEG3 inhibited proliferation and promoted apoptosis of BCa cells by regulating miR-96/TPM1 (Liu et al., 2018b). In addition, Huang et al. (2019) revealed that MEG3 negatively regulates the PHLPP2-c-Jun-c-Myc axis by competing with miR-27a, thus inhibiting the aggressiveness and metastasis of BCa cells. A detailed study demonstrated that MEG3 regulates the expression of PTEN by targeting miR-494 to inhibit the occurrence and development of BCa (Shan et al., 2020).

Therefore, MEG3 has an anti-tumor effect in BCa and could potentially provide alternative treatment options. Whereas, there are few studies on the role of MEG3 as a molecular sponge in BCa and future studies can focus on exploring whether other pathways or miRNAs play an antitumor role in BCa.

#### 2.3.4 MEG3 in prostate cancer

A serious threat to men’s health, PCa is one of the most prevalent malignancies of the genitourinary system; therefore, it is of great significance to understand its molecular mechanisms and search for new biomarkers to reduce PCa incidence.

A recent study pointed out that overexpression of MEG3 induced G0/G1 phase arrest by downregulating cyclin D1 expression in PC3 and DU145 cells, thus inducing tumor cell apoptosis in PCa (Luo et al., 2015). In addition, Wu et al. (2019) showed that MEG3 acts as a miRNA sponge in PCa and effectively inhibited PCa development by targeting miR-9-5p/QKI-5 axis. The *in vitro* model established by Tai et al. (2020) confirmed that PAMAM-PEG-EPDT3/pMEG3 nanoparticles improves the effect of gene therapy in castration-resistant PCa cells. In the same year, another group revealed that MEG3 inhibits PCa progression by binding to EZH2 and promoting H3K27 trimethylation of engrailed homeobox 2 (EN2) (Zhou et al., 2020). In summary, the above data suggest that MEG3 has a tumor-suppressive effect on PCa.

#### 2.3.5 MEG3 in testicular germ cell tumor

TGCT is a urogenital malignancy, in men aged 15–44 years, in the United States and is histologically classified as seminoma, non-seminoma, and spermatocytic neoplasms (Ghazarian et al., 2017). Although most patients with TGCT respond well to treatment, some respond poorly during the advanced stages; therefore, novel treatments could mitigate this issue.

A study to better understand the related molecular mechanisms clarified that a high level of MEG3 expression could competitively bind to miR-1297 and eliminate the inhibitory effect of miR-1297 on PTEN/PI3K/AKT (Yang et al., 2016). This leads to the inactivation of AKT and inhibits the growth of tumor cells. In conclusion, these findings indicate that MEG3 may be a promising target for TGCT diagnosis and treatment.

#### 2.3.6 MEG3 in breast cancer

Although predominant in women, BC comprises 31% of cancers, and its incidence has risen 41% since 1989, partly due to declining fertility rates and weight gain (Siegel et al., 2022). Therefore, a new therapeutic approach is required to elucidate the underlying molecular mechanisms of BC.

Sun et al. (2016a) showed that ectopic expression of MEG3 reduced MDM2 transcription and suppressed the proliferation, migration, and invasion of BC cells. Subsequent studies showed that the expression level of MEG3 in BC tissues is dramatically decreased (Zhang et al., 2016a). The serum level of MEG3 is related to BC differentiation, TNM stage, and lymph node metastasis. Other research showed that excessive MEG3 expression inhibited the growth, invasion, and tumor angiogenesis of BC by downregulating the AKT signaling pathway (Zhang et al., 2017b). In addition, Cui et al. (2018b) revealed that suppression of MEG3 expression in BC was positively correlated with the expression of heparin sulfate proteoglycan 2. A group from Zhengzhou University discovered that miR-506 regulated the expression of Sp1 transcription factor (SP1) and Sp3 transcription factor (SP3), thereby reducing MEG3 methylation and inhibiting the migration and invasion of BC cells (Wang et al., 2018b). Subsequently, Zhang et al. (2019c) suggested that MEG3 suppressed tumor growth and induced apoptosis of BC cells by activating the ER stress, NF-κB, and p53 pathways. A year later, another group firstly genotype three polymorphisms (rs3087918, rs11160608, and rs7158663) in MEG3 and found that the wild-type homozygous GG of MEG3 rs3087918 might be associated with a decreased risk of lung tumors (Ali et al., 2020). Recently, it was reported that the expression level of MEG3 was significantly decreased with the mutant A and G alleles, and the presence of both rs7158663 and low MEG3 is diagnostic or unfavorable prognostic factors for patients with BC (Zheng et al., 2020). Moreover, a team from Hebei University provided new insights into the molecular pathogenesis of triple-negative BC, where lncRNA MCM 3AP-AS1 promoted tumor cell proliferation by downregulating MEG3 (Ren et al., 2021). Shaker et al. (2021) showed that MEG3 is used as a miRNA decoy, leading to an increase in miR-182 and miR-29 expression.

MEG3 also plays a key role in radiotherapy and chemotherapy for BC. Bayarmaa et al. (2019) revealed that MEG3 rs10132552 was related to cisplatin-containing chemotherapy response in BC patients, and MEG3 rs10132552 and rs941576 might be associated with disease-free survival. Not long after, a study reported that inhibition of MEG3 methylation could weaken the chemotherapy resistance of BC cells to target genes; thus, playing a role in tumor suppression (Li et al., 2020b). Moreover, in another study, Yan et al. (2021) confirmed that cisplatin exerted an anti-tumor effect by activating the MEG3/NLR family pyrin domain containing 3 (NLRP3)/Caspase-1/gasdermin D (GSDMD) pathway in triple-negative BC.

Recent studies have also shown the presence of lncRNA-miRNA-mRNA networks in BC. An in-depth study revealed that MEG3 increases the expression of E-cadherin to inhibit tumor cell invasion and EMT by sponging miR-421 (Zhang et al., 2017c). Besides, Zhu et al. (2019) reported that overexpression of MEG3 inactivated the PI3K/AKT signal pathway by sponging miR-21 and suppressed the occurrence of BC. In subsequent studies, the authors found that MEG3 suppresses the pathogenesis of BC through miR-4513/phenazine biosynthesis, such as the protein domain containing (PBLD) axis (Zhu et al., 2020). Moreover, Dong et al. (2021) discovered a targeting relationship between MEG3, miR-141-3p, and RNA-binding motif single-stranded interacting protein 3 (RBMS3).

Hence, MEG3 can function as a tumor suppressor through increasing E-cadherin, PBLD and RBMS3 by inhibiting miR-421, miR-4513 and miR-141-3p respectively. Collectively, these data imply that MEG3 has an antitumor effect in BC.

#### 2.3.7 MEG3 in ovarian cancer

OC is a common malignancy of the female reproductive system. Due to the lack of clear early features and diagnostic biomarkers, many patients are diagnosed at a late stage, resulting in a high mortality rate (Niu et al., 2015). Although surgery, chemotherapy, and radiotherapy have some beneficial effects, the prognosis remains poor. Therefore, it is necessary to identify specific biomarkers for OC to elucidate its invasion and metastasis mechanisms.

A group from China Medical University revealed that MEG3 may be a potential biomarker for epithelial ovarian cancer (EOC) and acts as a tumor suppressor in EOC by modulating autophagy-related 3 (ATG3) activity and triggering autophagy (Xiu et al., 2017). Wang et al. (2018c) showed that high MEG3 expression may suppress tumor cell proliferation and boost apoptosis by targeting PTEN. Another study pointed out that MEG3 was decreased in OC tissues and was negatively related to AGAP2 antisense RNA 1 (AGAP2-AS1). AGAP2-AS1 could downregulate the expression of MEG3 and promote OC cell proliferation (Chen et al., 2019). In addition, a recent study proposed that the serum level of MEG3 is a marker of high-grade OC tumor progression (Buttarelli et al., 2020).

MEG3 also affects the chemosensitivity of patients with OC. A recent study showed that upregulated expression of MEG3 reduces the extracellular vesicle-mediated metastasis of miR-214 in OC cells; in turn, this reduces the resistance of recipient cells to drugs (Zhang et al., 2017d). Notably, these results revealed for the first time that curcumin can be used as a demethylating agent to restore MEG3 expression levels in OC. A subsequent study has shown that the level of MEG3 is used as a biomarker to predict cisplatin resistance of OC (El-Khazragy et al., 2020).

Recently, many studies have demonstrated that MEG3 exerts anti-cancer effects through the lncRNA-miRNA-mRNA axis. Wang et al. (2019b) stated that MEG3 may inhibit the occurrence and development of OC by regulating miR-219a-5p/EGFR axis. Not long after, a study demonstrated that MEG3 increased laminin subunit alpha 4 (LAMA4) expression by sponging miR-30e-3p, thereby suppressing OC cell growth, migration, and invasion (Liu et al., 2020). Moreover, Tao et al. (2020) demonstrated that upregulation of MEG3 plays a positive role by inhibiting proliferation and invasion, promoting cell apoptotic by targeting miR-205-5p.

Therefore, MEG3 may suppress the progress of OC through promoting the expression level of EGFR and LAMA4 by sponging miR-219a-5p and miR-30e-3p respectively. In conclusion, these studies showed that MEG3 is a useful biomarker for OC diagnosis and therapy.

#### 2.3.8 MEG3 in endometrial cancer

According to the US Department of Health and Human Services, EC is the sixth leading cause of cancer-related death. The number of new cases is increasing annually due to obesity and the aging population. Therefore, it is of great significance to study the molecular mechanisms, identify new biomarkers, and seek new diagnosis and treatment methods.

Guo et al. (2016) found that MEG3 expression was reduced in EC tissues, and that high expression of MEG3 inhibited the Notch signaling pathway in EC. Similar results indicated that MEG3 may inhibit the occurrence and development of EC through the Notch and PI3K pathways (Sun et al., 2017). In addition, 3 years later, another group demonstrated that the MEG3/miR-216a axis might mediate the inhibition of PD-L1 in invasive EC (Xu et al., 2020). Therefore, these data imply that MEG3 has an antitumor effect in EC.

#### 2.3.9 MEG3 in choriocarcinoma

Choriocarcinoma is a highly malignant tumor that occurs in women of childbearing age, and is rarely diagnosed in postmenopausal women or girls under 20 years (Rafanan et al., 2017). Therefore, there is an urgent need to develop treatment strategies for choriocarcinoma.

Ji and Li (2019) found that MEG3 inhibited choriocarcinoma formation by positively modulating miR-211/PI3K/AKT/AMPK signaling pathways. Similar results indicated that schisandrin A upregulated MEG3 expression and blocked the PI3K/AKT/NF-κB axis, thereby suppressing the migration and invasion of choriocarcinoma cells (Ji and Ma, 2020). Overall, these data verified that MEG3 acts as a tumor suppressor in choriocarcinoma.

#### 2.3.10 MEG3 in cervical cancer

The incidence of CC continues to increase due to extensive cytological screening, and it remains an ongoing public health problem (Siegel et al., 2022). Therefore, studying the molecular mechanisms, identifying new biomarkers, and seeking new diagnosis and treatment methods could benefit the population.

A study by Qin et al. (2013) showed that the expression of MEG3 was high in non-tumor tissue; it was considerably decreased in tumor tissues. Emerging evidence showed that decreased MEG3 expression was related to hypermethylation of the MEG3 promoter in CC tissues, leading to tumor recurrence and short OS (Zhang et al., 2017e). Subsequent studies have demonstrated that MEG3 methylation in plasma serves as a prognosticator or diagnostic tool for CC (Zhang et al., 2017f). Another study confirmed that MEG3 might bind p-STAT3 protein through ubiquitination to promote its degradation; thus, inhibiting proliferation and promoting the apoptosis of CC cells (Zhang and Gao, 2019). In addition, Wang et al. (2017b) further elaborated the role of MEG3 as a tumor suppressor in CC by modulating the PI3K/AKT/Bcl-2/Bax/P21 and PI3K/AKT/MMP-2/9 axes. An in-depth study revealed that the overexpression of MEG3 suppressed the survival, migration, and invasion of CC cells (Chen and Qu, 2018). Moreover, Wan and Zhao (2020) demonstrated that a low level of MEG3 was an independent prognostic factor for CC patients. Recently, it was demonstrated that MEG3 suppressed the development of CC cells and activated apoptosis by targeting miR-21 (Zhang et al., 2016b).

Research has also shown that MEG3 serves as a molecular sponge for miRNA to inhibit cancer as Zhu and Han (2019) found that lidocaine inhibits proliferation and promotes apoptosis of CC cells by modulating the MEG3/miR-421/anti-proligeration factor 1 (BTG1) axis. In addition, similar results confirmed that MEG3/miR-7-5p/stanniocalcin 1 (STC1) axis mediates ER stress and induces apoptosis in CC cells (Pan et al., 2021a).

In summary, the above data verified that MEG3 inhibited the occurrence and progression of CC. Still, some of the teams’ research had limitations, such as a lack of cell experiments to verify their conclusion. In addition, some studies on the molecular sponging mechanism of MEG3 have only explored the miRNAs where MEG3 acts, without further exploring the downstream pathways of miRNAs. Hopefully, these problems will be solved in the future.

It was demonstrated that MEG3 can inhibit the progress of genitourinary system neoplasms through increasing the expression of RASL11B, TPM1, PHLPP2, PTEN, QKI-5, PI3K/AKT, E-cadherin, PBLD, RBMS3, EGFR, PDGFRA, LAMA4, PD-L1, BTG1, and STC1 by sponging miR-7, miR-96, miR-27a, miR-494, miR-9-5p, miR-1297, miR-421, miR-4513, miR-141-3p, miR-219a-5p, miR-30e-3p, miR-216a, and miR-211, respectively. The specific mechanisms and functional characteristics of MEG3 in genitourinary system neoplasms are listed in Tables 3, 4.

**TABLE 3:** Characterization of MEG3 function in genitourinary system neoplasms.

Tumor types	Expression	Role	Function role	miRNAs	Related gene	Biomarker type	References
Renal cell carcinoma	Downregulation	Tumor suppressor	Proliferation, invasion, metastasis, and apoptosis	miR-7	RASL11B	Diagnostic	He et al. (2018)
Bladder cancer	Downregulation	Tumor suppressor	Proliferation and apoptosis	miR-96	TPM1	Diagnostic/Therapeutic	Liu et al. (2018b)
Bladder cancer	Downregulation	Tumor suppressor	Invasion and metastasis	miR-27a	PHLPP2-c-Jun-c-Myc	Prognostic/Therapeutic	Huang et al. (2019)
Bladder cancer	Downregulation	Tumor suppressor	Migration, invasion, and apoptosis	miR-494	PTEN	Diagnostic	Shan et al. (2020)
Prostate cancer	Downregulation	Tumor suppressor	Proliferation, invasion, metastasis, and apoptosis	miR-9-5p	QKI-5	Diagnostic	Wu et al. (2019)
Testicular germ cell tumor	Downregulation	Tumor suppressor	Proliferation	miR-1297	PTEN/PI3K/AKT	Therapeutic	Yang et al. (2016)
Breast cancer	Downregulation	Tumor suppressor	Apoptosis	miR-182, miR-29	—	Diagnostic/Prognostic	Shaker et al. (2021)
Breast cancer	Downregulation	Tumor suppressor	Proliferation and invasion	miR-421	E-cadherin	Therapeutic	Zhang et al. (2017c)
Breast cancer	Downregulation	Tumor suppressor	Proliferation, migration, invasion, apoptosis, and drug sensitivity	miR-4513	PBLD	Therapeutic	Zhu et al. (2020)
Breast cancer	Downregulation	Tumor suppressor	Proliferation and apoptosis	miR-141-3p	RBMS3	Diagnostic/Therapeutic	Dong et al. (2021)
Ovarian carcinoma	Downregulation	Tumor suppressor	Cell viability, proliferation, invasion, and migration	miR-219a-5p	EGFR	Diagnostic/Therapeutic	Wang et al. (2019b)
Ovarian carcinoma	Downregulation	Tumor suppressor	Proliferation, invasion, and migration	miR-30e-3p	LAMA4	Diagnostic/Therapeutic	Liu et al. (2020)
Ovarian carcinoma	Downregulation	Tumor suppressor	Cell viability, invasion, migration, and apoptosis	miR-205-5p	—	Diagnostic	Tao et al. (2020)
Endometrial carcinoma	Downregulation	Tumor suppressor	Migration and invasion	miR-216a	PD-L1	Diagnostic	Xu et al. (2020)
Choriocarcinoma	Downregulation	Tumor suppressor	Proliferation, migration, invasion, and apoptosis	miR-211	PI3K,AKT,AMPK	Therapeutic	Ji and Li, (2019)
Cervical carcinoma	Downregulation	Tumor suppressor	Proliferation and apoptosis	miR-21	—	Therapeutic	Zhang et al. (2016b)
Cervical carcinoma	Downregulation	Tumor suppressor	Cell viability and apoptosis	miR-421	BTG1	Therapeutic	Zhu and Han, (2019)
Cervical carcinoma	Downregulation	Tumor suppressor	Apoptosis	miR-7-5p	STC1	Therapeutic	Pan et al. (2021a)

**TABLE 4:** Features of the studies included in this review of genitourinary system neoplasms.

Study	Tumor types	Sample size (Normal: Tumor)	Detection Method	*p* value	TNM (*p* value)	LNM (*p* value)	DM (*p* value)	OS (*p* value)	References
Wang	Renal cell carcinoma	(29:29)	qRT-PCR	*p* < 0.05	—	—	—	—	Wang et al. (2015a)
He	Renal cell carcinoma	(72:72)	qRT-PCR	*p* < 0.01	—	—	—	—	He et al. (2018)
Teng	Wilms’ tumor	(54:54)	qRT-PCR	*p* < 0.05	—	*p* = 0.000	—	—	Teng et al. (2020)
Feng	Bladder cancer	(21:21)	qRT-PCR	*p* < 0.01	—	—	—	—	Feng et al. (2018)
Liu	Bladder cancer	(45:45)	qRT-PCR	*p* < 0.01	—	—	—	—	Liu et al. (2018b)
Huang	Bladder cancer	(27:27)	qRT-PCR	*p* < 0.05	—	—	*p* < 0.05	—	Huang et al. (2019)
Luo	Prostate cancer	(21:21)	qRT-PCR	*p* < 0.05	—	*p* = 0.2474	—	—	Luo et al. (2015)
Wu	Prostate cancer	(85:85)	qRT-PCR	*p* < 0.01	—	—	—	—	Wu et al. (2019)
Sun	Breast cancer	(20:20)	qRT-PCR	*p* < 0.05	—	—	—	—	Sun et al. (2016a)
Zhang	Breast cancer	(207:207)	qRT-PCR	*p* < 0.01	*p* = 0.011	*p* = 0.000	—	*p* < 0.001	Zhang et al. (2016a)
Zhang	Breast cancer	(165:33)	qRT-PCR	*p* < 0.05	—	—	—	—	Zhang et al. (2017b)
Ali	Breast cancer	(245:245)	qRT-PCR	*p* < 0.001	*p* = 0.001	—	—	—	Ali et al. (2020)
Zheng	Breast cancer	(434:700)	qRT-PCR	*p* = 0.046	—	—	—	—	Zheng et al. (2020)
Ren	Breast cancer	(60:60)	qRT-PCR	*p* < 0.05	—	—	—	—	Ren et al. (2021)
Li	Breast cancer	(374:374)	qRT-PCR	*p* < 0.001	*p* = 0.01	*p* = 0.012	—	—	Li et al. (2020b)
Zhang	Breast cancer	(90:90)	qRT-PCR	*p* < 0.05	*p* = 0.003	*p* = 0.012	—	*p* < 0.05	Zhang et al. (2017c)
Zhu	Breast cancer	(20:20)	qRT-PCR	*p* < 0.05	—	—	—	—	Zhu et al. (2019)
Zhu	Breast cancer	(31:31)	qRT-PCR	*p* < 0.05	—	—	—	—	Zhu et al. (2020)
Wang	Ovarian carcinoma	(100:30)	qRT-PCR	*p* < 0.05	—	—	*p* = 0.036	—	Wang et al. (2019b)
EI-Khazragy	Ovarian carcinoma	(317:317)	qRT-PCR	*p* < 0.05	—	—	*p* = 0.045	—	El-Khazragy et al. (2020)
Liu	Ovarian carcinoma	(30:10)	qRT-PCR	*p* < 0.001	—	—	—	—	Liu et al. (2020)
Tao	Ovarian carcinoma	(20:20)	qRT-PCR	*p* < 0.05	—	—	—	—	Tao et al. (2020)
Guo	Endometrial carcinoma	(30:30)	qRT-PCR	*p* < 0.01	—	—	—	—	Guo et al. (2016)
Sun	Endometrial carcinoma	(63:19)	qRT-PCR	*p* < 0.05	—	—	—	—	Sun et al. (2017)
Qin	Cervical carcinoma	(18:18)	qRT-PCR	*p* < 0.01	—	—	—	—	Qin et al. (2013)
Zhang	Cervical carcinoma	(72:72)	qRT-PCR	*p* < 0.05	—	*p* = 0.025	—	—	Zhang et al. (2017e)
Zhang	Cervical carcinoma	(168:168)	qRT-PCR	*p* = 0.045	—	*p* < 0.001	—	—	Zhang et al. (2017f)
Zhang	Cervical carcinoma	(22:22)	qRT-PCR	*p* < 0.05	—	—	—	—	Zhang and Gao, (2019)
Chen	Cervical carcinoma	(20:20)	qRT-PCR	*p* < 0.05	—	—	—	—	Chen and Qu, (2018)
Zhang	Cervical carcinoma	(108:108)	qRT-PCR	*p* < 0.01	—	*p* < 0.01	—	—	Zhang et al. (2016b)
Pan	Cervical carcinoma	(12:12)	qRT-PCR	*p* < 0.05	—	—	—	—	Pan et al. (2021a)

### 2.4 MEG3 in hematological system neoplasms

#### 2.4.1 MEG3 in leukemia

Leukemia is a malignant clonal hematopoietic stem cell disorder. It can be classified into acute myeloid leukemia (AML), acute lymphoblastic leukemia (ALL), chronic myeloid leukemia (CML), and chronic lymphoblastic leukemia (CLL). Therefore, it is important to investigate the molecular mechanisms underlying various types of leukemia and identify new therapeutic methods for effective treatment.

Some studies have shown the presence of lncRNA-miRNA-mRNA networks in leukemia. Yu et al. (2020b) analyzed that MEG3 regulates the expression of ALG9 by sponging miR-155, providing a new idea for the early diagnosis and chemotherapy resistance of AML. Similar results indicated that increased MEG3 expression modulated the expression of miR-147 to inhibit tumor cell proliferation and enhance apoptosis by modulating the JAK/STAT pathway (Li et al., 2018a).

Furthermore, Benetatos et al. (2010) suggested that MEG3 hypermethylation might be associated with decreased survival in patients with AML. Seven years later, another group confirmed that MEG3 expression is markedly reduced in AML cells (Yao et al., 2017a). And subsequent studies showed that hypermethylation of MEG3 promoter in AML might be related to downregulation of tet methylcytosine dioxygenase 2 (TET2) activity (Yao et al., 2017b). In addition, Sellers et al. (2019) highlighted the importance of the DLK1-MEG3 locus in the occurrence and development of AML. A recent study reported that the increase in MEG3 expression in ALL induction therapy is related to good prognosis and can improve patient survival (Gao, 2021). In CML, a study by Li et al. (2018b) showed that MEG3 interacted with miR-184 to downregulate related proteins and reduce cell proliferation and invasion. Additionally, a team from Southwest Medical University have contributed to our understanding of the mechanism of CLL resistance to imatinib (Zhou et al., 2017). It can alleviate imatinib resistance by regulating miR-21 to regulate the proliferation, apoptosis and expression of multidrug resistance transducers in leukemia cells. Moreover, another research group reported that MEG3 suppresses the growth of CML cells by sponging miR-21 and plays a regulatory role in the acute phase of CLL (Li et al., 2018c).

In summary, the above data verified that MEG3 inhibits the occurrence and progression of leukemia.

#### 2.4.2 MEG3 in multiple myeloma

MM is a malignant tumor accompanied by the secretion of monoclonal immunoglobulin, which can be detected in serum or urine (Röllig et al., 2015). Despite the adoption of innovative treatment strategies for MM, low survival rates have persisted. Thus, it is vital to study the molecular mechanisms underlying its pathogenesis and to discover new therapeutic strategies.

Emerging evidence shows a regulatory network of MEG3/miR-181a/homeobox A11 (HOXA11) in MM. Yu et al. (2020c) reported that the demethylation reagent 5-AZa-CdR might upregulate the level of p53 to suppress the proliferation of MM cells by upregulating MEG3 expression. Hence, these studies infer that MEG3 is a tumor suppressor in MM.

#### 2.4.3 MEG3 in T-cell lymphoblastic lymphoma

AS adult T-LBL has a poor prognosis and no internationally recognized therapy, it is pertinent to study its molecular mechanism and find new biomarkers, improving diagnosis and treatment methods.

The study by Fan et al. (2017) showed that MEG3 played a role as a tumor suppressor gene in T-LBL through miR-214/apoptosis inducing factor mitochondria associated 2 (AIFM2) pathways. Not long after, a study reported that MEG3 might suppress the invasion, migration, and drug resistance by suppressing the PI3K/mTOR axis in T-LBL cells (Deng et al., 2018). Together, these data support the hypothesis that MEG3 inhibits the occurrence and progression of T-LBL.

It was demonstrated that MEG3 can inhibit the progress of hematological system neoplasms through increasing the expression of ALG9, JAK/STAT, HOXA11, and AIFM2 by sponging miR-155, miR-147, miR-181a, and miR-214, respectively. The specific mechanisms and functional characteristics of MEG3 in hematological neoplasms are listed in Tables 5, 6.

**TABLE 5:** Characterization of MEG3 function in hematological, neuroendocrine, and other system neoplasms.

Tumor types	Expression	Role	Function role	miRNAs	Related gene	Biomarker type	References
Leukemia	Downregulation	Tumor suppressor	Proliferation and chemosensitivity	miR-155	ALG9	Diagnostic/Therapeutic	Yu et al. (2020b)
Leukemia	Downregulation	Tumor suppressor	Proliferation and metastasis	miR-184	—	Prognostic/Therapeutic	Li et al. (2018b)
Leukemia	Downregulation	Tumor suppressor	Proliferation and apoptosis	miR-147	JAK/STAT	Therapeutic	Li et al. (2018a)
Leukemia	Downregulation	Tumor suppressor	Proliferation, apoptosis, and chemosensitivity	miR-21	—	Diagnostic/Prognostic	Zhou et al. (2017)
Leukemia	Downregulation	Tumor suppressor	Proliferation and apoptosis	miR-21	—	Therapeutic	Li et al. (2018c)
Multiple myeloma	Downregulation	Tumor suppressor	Proliferation and apoptosis	miR-181a	HOXA11	Therapeutic	Shen et al. (2018)
Lymphoma	Downregulation	Tumor suppressor	Proliferation and apoptosis	miR-214	AIFM2	Prognostic/Therapeutic	Fan et al. (2017)
Glioma	Downregulation	Tumor suppressor	Proliferation, migration, and invasion	miR-19a	PTEN	Therapeutic	Qin et al. (2017)
Glioma	Downregulation	Tumor suppressor	Proliferation, migration, and invasion	miR-96-5p	MTSS1	Therapeutic	Zhang and Guo, (2019)
Glioma	Downregulation	Tumor suppressor	Migration and invasion	miR-377	PTEN	Diagnostic/Prognostic	Wang et al. (2019c)
Glioma	Downregulation	Tumor suppressor	Proliferation, migration, and EMT	miR-6088	SMARCB1	Therapeutic	Gong and Huang, (2020)
Meningioma	Downregulation	Tumor suppressor	Cell-cycle, migration, invasion, and proliferation	miR-29c	AKAP12	DiagnosticTherapeutic	Ding et al. (2020)
Thyroid carcinoma	Downregulation	Tumor suppressor	Proliferation and apoptosis	miR-182	—	Therapeutic	Liu et al. (2018a)
Pancreatic cancer	Downregulation	Tumor suppressor	Migration and invasion	miR-183	BRI3	Therapeutic	Zhang and Feng, (2017)
Pancreatic cancer	Downregulation	Tumor suppressor	Proliferation and migration	miR-374a-5p	PTEN	Therapeutic	Han et al. (2020)
Osteosarcoma	Downregulation	Tumor suppressor	Proliferation, migration, and invasion	miR-361-5p	FoxM1	Diagnostic/Therapeutic	Shen et al. (2019)
Osteosarcoma	Downregulation	Tumor suppressor	Proliferation, migration, and apoptosis	miR-184	—	Therapeutic	Li et al. (2020c)
Osteosarcoma	Downregulation	Tumor suppressor	Migration	miR-664a	—	Diagnostic/Therapeutic	Sahin et al. (2017)
Melanoma	Downregulation	Tumor suppressor	Proliferation, migration, invasion, and apoptosis	miR-499-5p	CYLD	Therapeutic	Long and Pi, (2018)
Melanoma	Downregulation	Tumor suppressor	Growth and metastasis	miR-21	E-cadherin	Therapeutic	Wu et al. (2020)

**TABLE 6:** Features of the studies included in this review of hematological, neuroendocrine, and other system neoplasms.

Study	Tumor types	Sample size (Normal: Tumor)	Detection method	*p* Value	TNM (*p* value)	LNM (*p* value)	DM (*p* value)	OS (*p* value)	References
Benetatos	Leukemia	(85:85)	qRT-PCR	*p* < 0.05	—	—	—	*p* = 0.04	Benetatos et al. (2010)
Yao	Leukemia	(29:20)	qRT-PCR	*p* < 0.05	—	—	—	—	Yao et al. (2017a)
Yao	Leukemia	(26:20)	qRT-PCR	*p* < 0.05	—	—	—	—	Yao et al. (2017b)
Gao	Leukemia	(117:117)	qRT-PCR	*p* < 0.001	—	—	—	*p* < 0.05	Gao, (2021)
Li	Leukemia	(57:57)	qRT-PCR	*p* < 0.01	—	—	—	—	Li et al. (2018b)
Li	Leukemia	(60:10)	qRT-PCR	*p* < 0.05	—	—	—	—	Li et al. (2018a)
Zhou	Leukemia	(68:68)	qRT-PCR	*p* < 0.05	—	—	—	—	Zhou et al. (2017)
Li	Leukemia	(40:10)	qRT-PCR	*p* < 0.05	—	—	—	—	Li et al. (2018c)
Shen	Multiple myeloma	(351:22)	qRT-PCR	*p* < 0.05	—	—	—	*p* = 0.002	Shen et al. (2018)
Yu	Multiple myeloma	(39:39)	qRT-PCR	*p* < 0.05	—	—	—	—	Yu et al. (2020c)
Fan	Lymphoma	(50:38)	qRT-PCR	*p* < 0.01	—	—	—	—	Fan et al. (2017)
Tang	Neuroblastoma	(6:6)	qRT-PCR	*p* < 0.01	—	—	—	—	Tang et al. (2016)
Zhuo	Neuroblastoma	(393:812)	qRT-PCR	*p* < 0.05	—	—	—	—	Zhuo et al. (2018)
Zhao	Glioma	(79:79)	qRT-PCR	*p* < 0.001	—	—	—	—	Zhao et al. (2018)
Xu	Glioma	(955:955)	qRT-PCR	*p* < 0.05	—	—	—	*p* < 0.05	Xu et al. (2021b)
Zhang	Glioma	(30:30)	qRT-PCR	*p* < 0.05	—	—	—	—	Zhang and Guo, (2019)
Ding	Meningioma	(32:5)	qRT-PCR	*p* < 0.05	—	—	—	—	Ding et al. (2020)
Gao	Retinoblastoma	(63:63)	qRT-PCR	*p* < 0.001	—	—	—	—	Gao and Lu, (2016)
Gao	Retinoblastoma	(63:63)	qRT-PCR	*p* < 0.001	—	—	—	—	Gao et al. (2017)
Gao	Retinoblastoma	(63:63)	qRT-PCR	*p* < 0.001	—	—	—	—	Gao et al. (2020)
Wang	Thyroid carcinoma	(16:16)	qRT-PCR	*p* < 0.05	—	—	—	—	Wang et al. (2015b)
Liu	Thyroid carcinoma	(20:20)	qRT-PCR	*p* < 0.05	—	—	—	—	Liu et al. (2018a)
Gu	Pancreatic cancer	(30:30)	qRT-PCR	*p* < 0.05	—	—	—	—	Gu et al. (2017)
Ma	Pancreatic cancer	(25:25)	qRT-PCR	*p* < 0.05	—	—	—	—	Ma et al. (2018)
Shen	Osteosarcoma	(204:204)	qRT-PCR	*p* < 0.05	*p* = 0.013	—	*p* = 0.02	—	Shen et al. (2019)
Li	Osteosarcoma	(18:18)	qRT-PCR	*p* < 0.05	—	—	—	—	Li et al. (2020c)
Tian	Osteosarcoma	(64:64)	qRT-PCR	*p* < 0.05	—	—	*p* = 0.011	*p* < 0.05	Tian et al. (2015)
Long	Melanoma	(42:42)	qRT-PCR	*p* < 0.05	*p* = 0.005	*p* = 0.000	*p* = 0.000	—	Long and Pi, (2018)
Wu	Melanoma	(25:25)	qRT-PCR	*p* < 0.05	—	—	—	—	Wu et al. (2020)

### 2.5 MEG3 in neuroendocrine system neoplasms

#### 2.5.1 MEG3 in neuroblastoma

NB is the most common extracranial solid tumor in children, with 90% occurring in children under 10 years of age. It is a neuroendocrine tumor its pathogenesis is still unclear.

An in-depth study revealed that MEG3, hyperpolarization-activated cyclic nucleotide-gated potassium channel 3 (HCN3), and linc01105 affect the proliferation and apoptosis of NB cells through the HIF-1α and p53 pathways (Tang et al., 2016). Additionally, Zhuo et al. (2018) proposed that MEG3 is a susceptibility gene for NB. The MEG3 polymorphism analysis indicated that subjects with the rs4081134 AG/AA genotype tended to develop NB in specific subgroups. Two years after, another group showed that high expression of MEG3 suppressed the development of NB by inhibiting FOXO1-mediated autophagy and mTOR-mediated EMT (Ye et al., 2020). In combination, these studies provide new insights into the disease.

#### 2.5.2 MEG3 in glioma

Gliomas are one of the most prevalent and aggressive brain neoplasms worldwide. It grows rapidly, has a strong invasion ability, and has a poor prognosis. Surgical treatment and postoperative chemoradiotherapy are the standard treatment for glioma however, chemotherapy resistance often leads to treatment failure. Therefore, it is imperative to study treatment strategies for gliomas.

A team from Jilin University reported that MEG3 inhibited the proliferation and migration of U251 cells by positive regulation of sirtuin 7 (Sirt7) and participates in the suppression of the PI3K/AKT/mTOR axis in glioma (Xu et al., 2018c). Another research group clarified that the decreased expression of MEG3 was closely related to the reduction in the OS of patients with glioma (Zhao et al., 2018). Subsequently, Buccarelli et al. (2020) stated that MEG3 inhibited the growth of glioma tumors by modulating cell adhesion, EMT, and cell proliferation. Nonetheless, the molecular mechanism of MEG3 in glioma is still not clear. In addition, another research group revealed that downregulation of MEG3 induced EMT, migration, and invasion of glioma cells, and overexpression of MEG3 induced autophagy in glioma cells (Yang et al., 2020b). Similar results indicated that MEG3 might be used as a biomarker for glioma prognosis and as a promising immunotherapy biomarker (Xu et al., 2021b).

Recently, several studies have shown that MEG3 inhibits glioma development through lncRNA-miRNA-mRNA networks. Emerging evidence shows that MEG3 inhibits the proliferation, migration, and invasion of glioma cells by sponging miR-19a to upregulate the expression level of PTEN (Qin et al., 2017). Zhang and Guo (2019) found that the MEG3/miR-96-5p/MTSS I-BAR domain-containing (MTSS1) axis may be a possible approach for treating gliomas. Recently, it has been reported that MEG3 interacts with miR-377 and PTEN and MEG3 inhibited glioma cells through miR-377/PTEN signals (Wang et al., 2019c). In addition, similar results demonstrated that MEG3 glioma progression by sponging miR-6088 to target the SMARCB1 protein (Gong and Huang, 2020).

These data above suggest that MEG3 acts as a tumor suppressor in leukemia through the lncRNAs-miRNAs-mRNAs axis as well as a number of other pathways. In future studies, the researchers can refine their search for the exact molecular mechanism.

#### 2.5.3 MEG3 in meningioma

Among the most common primary tumors of the nervous system, meningioma originates from arachnoid meningeal epithelial cells. It is very important to understand the pathogenesis of meningiomas and identify effective treatment strategies.

Recently, a team from Fujian Medical University found that MEG3 mediated meningioma cell cycle arrest through miR-29c/A-kinase anchoring protein 12 (AKAP12) axis and inhibited tumor cell migration, invasion, and proliferation; thus, providing a new biomarker for the treatment of meningioma (Ding et al., 2020). In summary, this finding implies that MEG3 is a tumor suppressor in meningioma.

#### 2.5.4 MEG3 in retinoblastoma

Although RB is the most common form of intraocular malignancy in both infants and young children, its occurrence is rare (Cassoux et al., 2017). RB severely damages children’s visual function and life; therefore, new approaches to molecular regulatory mechanisms are urgently needed.

Gao and Lu (2016) demonstrated that decreased MEG3 expression is related to RB severity. MEG3 suppresses tumor growth by inhibiting the Wnt/β-catenin pathway. One year later, they clarified that MEG3 was epigenetically inactivated because of the abnormal hypermethylation of its promoter (Gao et al., 2017). Studies have shown that overexpression of DNMT1 can induce MEG3 gene promoter methylation and inactivation; thus, promoting RB cell proliferation (Gao et al., 2020). In conclusion, these data imply that MEG3 inhibits RB progression and functions as a diagnostic and therapeutic target for RB.

#### 2.5.5 MEG3 in thyroid cancer

In adults, TC is the most prevalent primary endocrine malignant neoplasm, and new studies on its molecular regulatory mechanisms are necessary.

An in-depth study showed that high MEG3 expression inhibited tumor invasion and migration by targeting Rac1 (Wang et al., 2015b). A subsequent study clarified that MEG3 enhances the radiosensitivity of TC cells to ^131^I by sponging miR-182 and inducing tumor cell apoptosis (Liu et al., 2018a). Still, this study lacks elaboration of the detailed mechanisms of how MEG3 affects ^131^I radiation sensitivity. In summary, the findings indicated that MEG3 plays a significant role in inhibiting TC cell growth.

#### 2.5.6 MEG3 in pancreatic cancer

PC is a rare tumor that originates from islet cells; It is highly malignant and has a very poor prognosis. In terms of treatment, PC is fairly resistant to most conventional treatment regimens. Therefore, the development of new treatment strategies is vital.

Modali et al. (2015) suggested that MEG3 activation and/or inactivation through epigenetic modification might have therapeutic effects on PC. A year later, a team from Soochow University showed that fenofibrate inhibits PC cell proliferation by upregulating MEG3-mediated p53 activation (Hu et al., 2016). Not long after, a study reported that MEG3 regulates the PI3K/AKT/Bcl-2/Bax/Cyclin D1/p53 as well as PI3K/AKT/MMP-2/MMP-9 signaling pathways in PC to suppress tumor growth (Gu et al., 2017). Another study highlighted that MEG3 is downregulated in PC; their study comprehensively established the epigenetic mechanism of MEG3 regulation of c-MET (Iyer et al., 2017). In addition, Zhang and Feng (2017) confirmed that MEG3 might play a tumor-suppressive role in PC by sponging miR-183 and targeting the brain protein I3 (BRI3). Subsequent studies showed that MEG3 exerts tumor-inhibitory effects by regulating cell proliferation, migration, invasion, EMT, and chemotherapy sensitivity (Ma et al., 2018). Further, Han et al. (2020) reported that MEG3 sponges miR-374a-5p to modulate the expression level of PTEN and suppress tumor cell growth in PC. According to another research group, the expression level of MEG3 was negatively related to the expression of PI3K, which was closely related to tumor size, metastasis, and vascular infiltration (Pan et al., 2021b). These results indicate that MEG3 is an effective target for the diagnosis and treatment of PC.

It was demonstrated that MEG3 can inhibit the progress of neuroendocrine system neoplasms through increasing the expression of PTEN, MTSS1, SMARCB1, AKAP12, Rac1, and BRI3 by sponging miR-19a, miR-96-5p, miR-377, miR-6088, miR-29c, miR-182, miR-183, and miR-374a-5p, respectively. The specific mechanisms and functional characteristics of MEG3 in neuroendocrine system neoplasms are listed in Tables 5, 6.

### 2.6 MEG3 in other neoplasms

#### 2.6.1 MEG3 in osteosarcoma and chordoma

Osteosarcomas account for the majority of bone tumors in children and adolescents. Moreover, the incidence of secondary malignancy in patients is 3.7 times higher than that in the normal population, mainly due to the adjuvant chemotherapy used in osteosarcoma (Bagcchi, 2014). Chordoma is a rare bone tumor with a high recurrence rate; however, its pathogenesis remains unclear. Therefore, new treatment methods are essential.

Two years ago, a team from Nanjing medical university stated that MEG3 contributes greatly to the diagnosis and treatment of osteosarcoma by sponging miRNAs (Jiang et al., 2019). Not long after, another team confirmed that the MEG3/miR-361-5p/forkhead box M1 (FoxM1) signaling axis may be a diagnostic biomarker or therapeutic target for osteosarcoma (Shen et al., 2019). In addition, a study by Li et al. (2020c) proved that MEG3 regulates the proliferation, migration, and apoptosis of osteosarcoma cells by targeting miR-184 and Wnt/β-catenin.

Emerging evidence reported that MEG3 knockdown was an independent prognostic biomarker affecting the OS of patients with osteosarcoma (Tian et al., 2015). Subsequently, Sun et al. (2016b) found that Ewing sarcoma associated transcript 1 (EWSAT1) played a vital role in the growth and metastasis of osteosarcoma cells, through modulating the expression level of MEG3. Another study observed that the MEG3/miR-644a axis may be a novel factor for the diagnosis and treatment of osteosarcoma (Sahin et al., 2017). The results of miR-664a inhibition assay showed that up-regulation of miR-664a inhibits MEG3 gene expression in osteosarcoma. In addition, another research group demonstrated that MEG3 might inhibit the development and metastasis of osteosarcoma by inhibiting the Notch and TGFβ signaling pathways (Zhang et al., 2017g). Shi et al. (2018) revealed that upregulation of MEG3 significantly increased the transactivation of p53, modulated the expression of downstream proteins, and suppressed the proliferation, invasion, and migration of tumor cells. Recently, similar results indicated that MEG3 might effectively suppress the development of osteosarcoma cells and boost cell apoptosis by inhibiting the activation of the intracellular Notch signaling pathway (Chen et al., 2020).

A recent study demonstrated that increased MEG3 expression inhibited the proliferation of chordoma cells, revealing the role of the imprinted gene cluster DLK1-MEG3 in the occurrence and development of chordoma (Chen et al., 2017).

Therefore, these studies imply that MEG3 is a tumor suppressor in osteosarcoma and chordoma. Whereas, if some of the researchers can add animal experiments to confirm their conclusion, it will be even more convincing. And the study of the exact molecular mechanism is very necessary in the future.

#### 2.6.2 MEG3 in melanoma

Melanoma is a highly malignant tumor, and its incidence is gradually increasing. Although it can be treated by surgical resection in the early stage, if it has spread to a metastatic stage, it is almost impossible to cure (Gray-Schopfer et al., 2007). Hence, new treatment strategies would be beneficial.

Li et al. (2018d) suggested that MEG3 might inhibit melanoma progression by activating the Wnt signaling pathway. In addition, another team showed that MEG3 modulated the expression of CYLD by targeting miR-499 and suppressed the proliferation and invasion of melanoma (Long and Pi, 2018). Two years later, a research group from Sun Yat-sen University revealed that MEG3 might inhibit tumor growth and metastasis by modulating the miR-21-E-cadherin axis (Wu et al., 2020).

It was demonstrated that MEG3 can inhibit the progress of other system neoplasms through increasing the expression of FoxM1, CYLD, and E-cadherin by sponging miR-361-5p, miR-499-5p, and miR-21, respectively. The specific mechanisms and functional characteristics of MEG3 in other systemic neoplasms are listed in Tables 5, 6.

### 2.7 MEG3 in various common inflammatory processes

**FIGURE 2:**
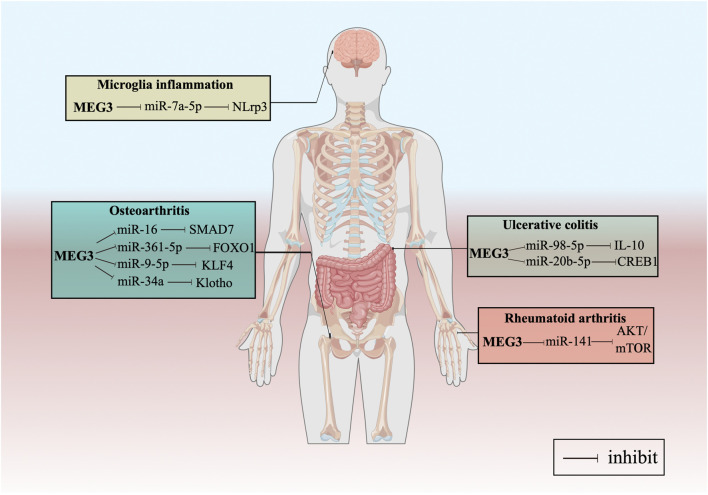
MEG3 mediates mechanisms involved in various common inflammatory processes.

Furthermore, we have summarized the regulatory pathways of MEG3 in various common inflammatory processes. Xu and Xu (2017) found that the expression level of MEG3 decreased in cartilage tissues and that the downregulation of MEG3 led to the osteoarthritis (OA) through the miR-16/SMAD7 axis. Similar results indicated that MEG3 promotes cell proliferation and inhibits cell apoptosis and extracellular matrix degradation through the miR-361-5p/FOXO1 pathway in OA (Wang et al., 2019d). Recently, it was reported that MEG3 induces the expression of KLF4 by sponging miR-9-5p, promoting chondrocyte proliferation and migration, and inhibiting inflammation (Huang et al., 2021). Additionally, Xiong et al. (2022) found that MEG3 inhibits the progression of OA by regulating the miR-34a/Klotho axis. A group from Jining Medical University showed that MEG3 might exert anti-inflammatory effects by sponging miR-146a in ankylosing spondylitis (Li et al., 2020d). In another study, MEG3 could act as a molecular sponge for miR-7a-5p to regulate the expression level of NOD-like receptor 3 (Meng et al., 2021). Moreover, Wang et al. (2021c) stated that MEG3 might regulate IL-10 expression by targeting miR-98-5p, which may lead to its protective effect against ulcerative colitis (UC). Another study showed how MEG3 alleviated UC inflammation *via* the miR-206-5p/CREB1 pathway (Wang et al., 2021d). Finally, Li et al. (2019c) reported that MEG3 may inhibit rheumatoid arthritis through the miR-141/AKT/mTOR signaling pathway (Figure 2).

## 3 Common mechanisms of MEG3 in various cancers

### 3.1 MEG3 functions as a tumor suppressor by regulating common cell signal transduction pathways

MEG3 can play the role of tumor suppressor in many tumors by regulating the p53 pathway, PTEN/PI3K/AKT pathway, Wnt/β-catenin pathway, JAK/STAT pathway and other common cell signal transduction pathways. Yu et al. (2020c) showed that the demethylation reagent 5-AZa-CdR might increase the expression level of p53 to suppress the proliferation of MM cells by upregulating MEG3 expression. Pu et al. (2019) identified that MEG3 inhibited autophagy by downregulating ILF3 and regulating the PI3K-Akt-mTOR and Beclin-1 signaling pathways. Liu et al. (2017) demonstrated that the expression of MEG3 was downregulated and negatively regulated the Wnt/β-catenin signaling pathway to inhibit the proliferation and metastasis of oral squamous cell carcinoma. Besides, Tan et al. (2019) showed that high expression of MEG3 promotes the expression of SOCS5 and SOCS6 *via* the miR-548d-3p-JAK-STAT axis in oral cancer.

### 3.2 MEG3 and miRNAs

#### 3.2.1 MEG3 interacts with miRNAs as competitive endogenous RNA

It is well known that miRNA can lead to gene silencing by binding mRNA, while MEG3 can function as a ceRNA to affect gene silencing caused by miRNA by binding to miRNA through microRNA response elements, thereby upregulating the expression of target genes. This mechanism is involved in almost all tumor types in which MEG3 acts.

Peng et al. (2015) reported that MEG3 upregulated Bcl-2 by competitively binding to miR-181a, thus suppressing the proliferation, migration, and invasion of GC cells. Wu et al. (2021) showed that the MEG3/miR-544b/BTG2 axis may play a crucial role in the occurrence and progression of HCC. Wu et al. (2019) showed that MEG3 acts as a miRNA sponge in PCa and effectively inhibited PCa development by targeting miR-9-5p/QKI-5 axis. Zhang et al. (2017c) found that MEG3 increases the expression of E-cadherin to inhibit tumor cell invasion and EMT by sponging miR-421. In addition, Pan et al. (2021a) confirmed that MEG3/miR-7-5p/STC1 axis mediates ER stress and induces apoptosis in CC cells. Li et al. (2018a) found that increased MEG3 expression modulated the expression of miR-147 to inhibit tumor cell proliferation and enhance apoptosis by modulating the JAK/STAT pathway. Shen et al. (2018) found a regulatory network of MEG3/miR-181a/HOXA11 in MM. Qin et al. (2017) showed that MEG3 inhibits the proliferation, migration, and invasion of glioma cells by sponging miR-19a to upregulate the expression level of PTEN. Han et al. (2020) reported that MEG3 sponges miR-374a-5p to modulate the expression level of PTEN and suppress tumor cell growth in PC. Shen et al. (2019) confirmed that the MEG3/miR-361-5p/FoxM1 signaling axis may be a diagnostic biomarker or therapeutic target for osteosarcoma. Wu et al. (2020) revealed that MEG3 might inhibit tumor growth and metastasis in melanoma by modulating the miR-21-E-cadherin axis.

#### 3.2.2 Direct effect of MEG3 and miRNAs

Zhang et al. (2018) suggested that MEG3 may regulate the proliferation and metastasis of oral squamous cell carcinoma cells by targeting miR-21. Zhou et al. (2017) hypothesized that high expression of MEG3 reversed imatinib resistance, inhibited tumor cell development and increased apoptosis by targeting the expression of miR-21.

### 3.3 MEG3 functions as a tumor suppressor by increasing the sensitivity of radiotherapy or chemotherapy

Many studies have shown that MEG3 can regulate the processes related to drug resistance. Gao et al. (2021) showed that MEG3 enhances the anti-tumor activity of curcumin in gemcitabine-resistant NSCLC cells through the PTEN pathway. A study by El-Khazragy et al. (2020) revealed that the level of MEG3 is used as a biomarker to predict cisplatin resistance of OC. Liu et al. (2018a) clarified that MEG3 enhances the radiosensitivity of TC cells to ^131^I by sponging miR-182 and inducing tumor cell apoptosis.

### 3.4 MEG3 and methylation modification

Studies have confirmed that hypermethylation of the MEG3 promoter CpG island is caused the decrease or deletion of MEG3 gene expression level was the main reason. Wu et al. (2021) showed that m6A-induced MEG3 may inhibit the proliferation, migration and invasion *via* the MEG3/miR-544b/BTG2 axis in HCC. Yu et al. (2020c) reported that the demethylation reagent 5-AZa-CdR might upregulate the level of p53 to suppress the proliferation of MM cells by upregulating MEG3 expression. Studies have shown that overexpression of DNMT1 can lead to MEG3 gene promoter methylation and inactivation, which will promote RB cell proliferation (Gao et al., 2020).

In conclusion, the expression of MEG3 is downregulated or absent in a variety of tumors. Overexpression of MEG3 may affect and regulate common cancer-related pathways and interact with miRNAs, mRNAs and proteins through transcriptional regulation, post-transcriptional regulation or epigenetic regulation to play a tumor suppressor role. In the interaction with miRNA, MEG3 can regulate multiple miRNAs, and MEG3 plays a major role in tumor suppression as a ceRNA. Among the tumor suppressive effects of MEG3, the most common is that MEG3 down-regulates the downstream miRNA through the effect of molecular sponge, thus upregulates the expression of its target genes. Target genes can activate common cancer-associated pathways to play the role of tumor suppressor in various tumors. For each type of tumor, MEG3 can exert its effects through multiple pathways.

## 4 Conclusion and future perspectives

Cancer poses a severe threat to human health globally. Although the overall mortality rate of cancer has declined in recent years, its early diagnosis and treatment still face major challenges. Many patients with malignant tumors are diagnosed at a late stage and have a poor prognosis. Therefore, it is imperative to search for novel biomarkers and explore the various molecular mechanisms for early diagnosis and treatment of cancer.

Numerous studies have shown that lncRNAs are abnormally expressed in many diseases and can act as tumor suppressors or oncogenes. The expression of MEG3 in various tumor tissues and cells is lower than that in normal adjacent tissues. Thus, the loss of MEG3 expression is associated with tumorigenesis. MEG3 overexpression suppresses tumor cell growth in many neoplasms, including squamous cell carcinoma of the head and neck, lung cancer, ESCC, GC, HCC, GBC, CCA, CRC, RCC, WT, BCa, PCa, TGCT, BC, OC, EC, choriocarcinoma, CC, leukemia, MM, T-LBL, neuroblastoma, glioma, meningioma, RB, TC, PC, osteosarcoma, chordoma, and melanoma. Abnormal expression of MEG3 in tumors is closely related to a variety of clinical and pathological features such as age, tumor size, vascular invasion, distant metastasis, OS, and neoplasm recurrence. Many studies have shown that the upregulation of MEG3 may inhibit the proliferation, migration, and invasion of tumor cells and promote the apoptosis.

MEG3 not only has many downstream molecular pathways but also some upstream regulators. Fan et al. (2019) found that arsenic trioxide inhibits EMT in HCC, partly by upregulating MEG3 expression. Wu et al. (2021) disclosed that METTL3-mediated N6-methyladenosine modification might decrease MEG3 expression, which is critical for the occurrence and progression of HCC. Zhu et al. (2018) revealed that vitamin D can induce MEG3 expression, thereby inhibiting CRC cell proliferation and migration. Ji and Ma (2020) showed that schisandrin A might suppress the growth of choriocarcinoma cells by upregulating the expression of MEG3. Gao et al. (2020) explained that DNMT1 can promote the proliferation of RB cells by silencing the expression of MEG3.

**FIGURE 3:**
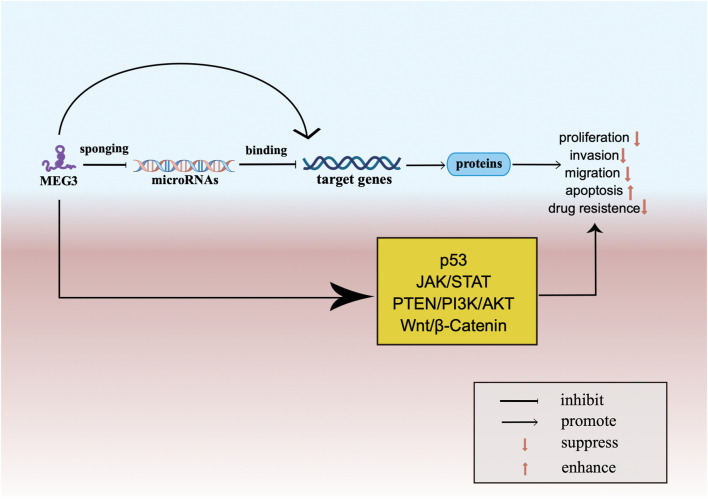
MEG3 mediates mechanisms involved in various cancers.

In the review, as described in Figure 3, we summarized the specific mechanism by which MEG3 acts as a tumor suppressor. Firstly, as described in Tables 1–6, MEG3 can inhibit the occurrence, development, and improve the prognosis of tumors by directly interacting with miRNA or acting on target genes and proteins. Second, MEG3 acts as a molecular sponge for miRNAs and as a tumor suppressor through the MEG3-miRNA-mRNA axis. In addition, we found that upregulation of MEG3 expression increases the sensitivity of tumor cells to radiotherapy and chemotherapy; thus, improving the effectiveness of current treatment strategies.

In almost all reports, the expression of MEG3 in tumor tissues is reduced or absent compared with adjacent normal tissues, and overexpression of MEG3 in tumors can play a role in tumor inhibition, such as inhibiting tumor proliferation, migration, invasion or promoting tumor cell apoptosis. These reports have shown that MEG3 is a novel target for tumor diagnosis and therapy. However, some studies failed to draw positive conclusion because of insufficient sample size. Therefore, future studies with larger sample sizes are required to elucidate the specific functional mechanism. At present, research on MEG3 still requires in-depth discussion, and its specific mechanism of action in tumors must be further investigated.

## Glossary

LncRNA: Long non-coding RNA
MEG3: Maternally expressed gene 3
BC: Breast cancer
PCa: Prostate cancer
CRC: Colorectal cancer
UCA1: Urothelial cancer associated 1
ZNF24TR: Zinc finger protein 24 transcription regulator
PC: Pancreatic cancer
ATF3: Activating transcription factor 3
ZNF24: Zinc finger protein 24
VEGFA: Vascular endothelial growth factor A
ESCC: Esophageal squamous cell carcinoma
GC: Gastric cancer
HCC: Hepatocellular carcinoma
GBC: Gallbladder cancer
CCA: Cholangiocarcinoma
RCC: Renal cell cancer
WT: Wilm’s tumor
BCa: Bladder cancer
TGCT: Testicular germ cell tumor
OC: Ovarian cancer
EC: Endometrial cancer
CC: Cervical cancer
MM: Multiple myeloma
T-LBL: T-cell lymphoblastic lymphoma
RB: Retinoblastoma
TC: Thyroid cancer
NPC: Nasopharyngeal carcinoma
EMT: Epithelial to mesenchymal transition
APAF-1: Apoptotic peptidase activating factor 1
NSCLC: Non-small cell lung cancer
PTEN: Phosphatase and tensin homolog
SOX7: SRY-box transcription factor 7
Skp2: S-phase kinase associated protein 2
SLC34A2: Solute carrier family 34 member 2
DKC1: Dyskerin pseudouridine synthase 1
FOXO1: Forkhead box O1
ER: Endoplasmic reticulum
PSAT-1: Phosphoserine aminotransferase 1
GSK-3β: Glycogen synthase kinase 3 beta
FOXP3: Forkhead box P3
DNMT-1: DNA methyltransferase 1
E2F3: E2F transcription factor 3
SFRP1: Secreted frizzled related protein 1
uHRF1: Ubiquitin like with PHD and ring finger domains 1
AP1G1: Adaptor related protein complex 1 subunit gramma 1
ILF3: Interleukin enhancer binding factor 3
DNMT3B: DNA methyltransferase 3 beta
ADH4: Alcohol dehydrogenase 4
ERp29: Endoplasmic reticulum protein 29
SOX11: SRY-box transcription factor 11
MMP-2: Matrix metallopeptidase 2
MMP-9: Matrix metallopeptidase 9
LATS2: Large tumor suppressor kinase 2
TRAF3: TNF receptor associated factor 3
SPHK1: Sphingosine kinase 1
PDCD4: Programmed cell death 4
PDHB: Pyruvate dehydrogenase E1 subunit beta
RASL11B: RAS like family 11 member B
EN2: Engrailed homeobox 2
SP1: Sp1 transcription factor
SP3: Sp3 transcription factor
NLRP3: NLR family pyrin domain containing 3
GSDMD: gasdermin D
PBLD: Phenazine biosynthesis like protein domain containing
RBMS3: RNA binding motif single stranded interacting protein 3
EOC: Epithelial ovarian cancer
ATG3: Autophagy related 3
AGAP2-AS1: AGAP2 antisense RNA 1
PDGFRA: Platelet derived growth factor receptor alpha
BTG1: Anti-proligeration factor 1
STC1: Stanniocalcin 1
AML: Acute myeloid leukemia
ALL: Acute lymphocyte leukemia
CML: Chronic myelogenous leukemia
CLL: Chronic lymphocytic leukemia
TET2: Tet methylcytosine dioxygenase 2
HOXA11: Homeobox A11
AIFM2: Apoptosis inducing factor mitochondria associated 2
HCN3: Hyperpolarization activated cyclic nucleotide gated potassium channel 3
Sirt7: Sirtuin 7
MTSS1: MTSS I-BAR domain containing
AKAP12: A-kinase anchoring protein 12
BRI3: Brain protein I3
FoxM1: Forkhead box M1
EWSAT1: Ewing sarcoma asssociated transcript 1
OA: osteoarthritis
ECM: extracellular matrix
AS: Ankylosing Spondylitis
NLrp3: nod-like receptor 3
UC: ulcerative colitis
RA: rheumatoid arthritis
ceRNA: competitive endogenous RNA
